# A Multi-Criteria Index for Ecological Evaluation of Tropical Agriculture in Southeastern Mexico

**DOI:** 10.1371/journal.pone.0112493

**Published:** 2014-11-18

**Authors:** Esperanza Huerta, Christian Kampichler, Susana Ochoa-Gaona, Ben De Jong, Salvador Hernandez-Daumas, Violette Geissen

**Affiliations:** 1 El Colegio de la Frontera Sur, Unidad Campeche, Dpto. Agroecología, Campeche, México; 2 Universidad Juárez Autónoma de Tabasco, División de Ciencias Biológicas, Villahermosa, Tabasco, México; 3 Sovon Dutch Centre for Field Ornithology, Natuurplaza (Mercator 3), Nijmegen, The Netherlands; 4 El Colegio de la Frontera Sur, Unidad Campeche, Dpto. Sustainability Sciences, Campeche, México; 5 University of Bonn - INRES, Bonn, Germany; 6 Wageningen University and Research Center – Alterra, Wageningen, Gelderland, Netherlands; Southwest University, China

## Abstract

The aim of this study was to generate an easy to use index to evaluate the ecological state of agricultural land from a sustainability perspective. We selected environmental indicators, such as the use of organic soil amendments (green manure) versus chemical fertilizers, plant biodiversity (including crop associations), variables which characterize soil conservation of conventional agricultural systems, pesticide use, method and frequency of tillage. We monitored the ecological state of 52 agricultural plots to test the performance of the index. The variables were hierarchically aggregated with simple mathematical algorithms, if-then rules, and rule-based fuzzy models, yielding the final multi-criteria index with values from 0 (worst) to 1 (best conditions). We validated the model through independent evaluation by experts, and we obtained a linear regression with an *r^2^* = 0.61 (*p* = 2.4e-06, *d.f.* = 49) between index output and the experts’ evaluation.

## Introduction

In the past 60 years, degradation and deforestation of tropical forests worldwide have occurred much faster and more extensively than in any other period in history [1], [2]. Furthermore, countries like Mexico have been undergoing drastic land use changes. In the tropics of Mexico large parts of its lowland rainforest areas have been converted into pasture and cropland. In the state of Tabasco, for example, only 3.4% of the state is covered with original forest [3], whereas 76.4% of the surface was used for cattle production in 2000 [4] and 15.6% was used for agriculture, principally sugarcane and fruit plantations [3]. The ecological consequences of these land-use changes in Tabasco are well documented. Soil losses in hilly regions are very high up to 200 t ha^−1^ year^−1^ [5]; high pesticide and fertilizer inputs to crops that have replaced forests have caused considerable environmental contamination [6], [7], and soil fertility is decreasing [8], [5], [9].

It is of the utmost importance to identify sustainable land use strategies which are economically attractive for the region's farmers and which may also reconcile the need for food production with that of soil conservation. In order to assist a variety of stakeholders at the local and regional level in making land use decisions, simple evaluation tools are needed. This is even more needed since a high percentage of the population consists of immigrants from other Mexican states, who are unfamiliar with the conditions of the humid tropics and use intensive techniques for farming the land. This is mostly due to large-scale agricultural development projects, such as “Plan Balancán-Tenosique,” named after the two municipalities involved, in which, in the 1970 s, over 1100 km^2^ of lowland rain forest was destroyed and converted into crop and pasture land. Ecological values of the 1970 s were very different from those of the present, and government representatives were willing to deforest in order to grant land to farmers [10].

The direct impact of farming is difficult to measure due to methodological difficulties (impossibility of measurement, complexity of the system) or practical reasons (time, costs) [11]. Therefore, the use of indicators appears to be an alternative way of guiding land use decisions [12], [13]. However, the “indicator explosion” [14], that is, the use of an exaggerated number of indicators aimed at assessing environmental impacts of agricultural activities, has been of little use to local decision-makers. Particularly in the tropics, land use decisions are still based on the informal opinion of local experts rather than on implementation of Decision Support Systems for environmentally sound resource management [15]. On the one hand, this is due to farmers’ restricted access to modern communications and information technologies; on the other, application of indicators is often beyond the capability of local farmers. For example, the agricultural sustainability index proposed by Nambiar et al. [16], which aims to measure agricultural sustainability as a function of biophysical, chemical, economic, and social indicators, would require considerable training of government stakeholders and farmers in order to be applied, and such training is rarely available.

Thus, any tool which local farmers or regional decision makers may use to support their decision making must be as simple as possible. Agroecosystems (like any other ecosystem) are too complex to be precisely measured and evaluated [17]. We agree with the view of Darnhofer et al. [18] in favour of developing less precise rules of thumb which may be used by farmers as well as to guide local land-use decisions toward a more environmentally friendly system of agriculture.

This index may provide farmers and others involved with a tool to evaluate the sustainability of their management of crop land which is oriented toward diminishing soil damage and conserving soil fertility. The index, exclusively based on terms which describe the environmental conditions of the crop system, is accessible to most farmers, and may be calculated using a simple internet application. In this paper we present a simple, easy-to-use index in order to evaluate the ecological state of farms in south-eastern Mexico. We applied the indicator system to 52 crop production systems in south-eastern Mexico and compared the results of the indicator system with expert opinions.

Human knowledge of how to efficiently and sustainably manage complex systems (including agricultural systems) is incomplete and much of what is thought to be known about this topic is actually incorrect. Yet, decisions must be made by policy makers, agricultural extension agents, and farmers despite uncertainty and knowledge gaps [19]. Therefore, tools to support local decision-makers must be flexible, should not enter into too much detail or precision, and should allow for an adaptive strategy which promotes “learning through management” [19]. Consequently, our rationale for developing an index which aids farmers in making environmentally friendly land-use decisions is based on basic, simplified ecological concepts, i.e. the presence of trees, since trees within an agroecosystem enhance soil microclimate in terms of radiation partitioning (shading), evapotranspiration partitioning, and rain interception/redistribution [20]. These factors all help to retain soil moisture. Branches, bark, roots, and living and dead leaf surfaces provide shelter [21] for soil micro-, meso-, and macro-invertebrates. Tree cover for instance, enhances above- and below-ground diversity, serving to support agricultural sustainability [22].

## Materials and Methods

### 1. Rationale of index composition

We define conventional agriculture as a cropping system, typically promoted by government development programs, that is “capital-intensive, large-scale, highly mechanized agriculture with monocropping and extensive use of synthetic fertilizers, and pesticides” [23], [24]. Furthermore, we acknowledge that farming systems are sustainable only if “they minimize the use of external inputs and maximize the use of internal inputs already present on the farm” [25], [26]. The strategy most frequently linked to sustainability is reduction or elimination of agrochemicals, particularly chemical fertilizers and pesticides [25], [27], [28], [29], [30], [31]. Another key to sustained productivity of agricultural systems is the maintenance of soil functions, such as organic matter and nutrient cycling [32], based on organic inputs [33], above-and below-ground biodiversity [22], and diversifying crop systems with nitrogen-fixing legumes [34]. The principal role of the index we propose is to characterize methods of tillage, external inputs, and crop structure.

### 2. Primary indicators

We chose 12 field variables as primary indicators related to the above mentioned aspects of ecologically sound agricultural land use based on farmer’s practices. These are easy to evaluate in the field and characterize plot structure (primary indicators: tree cover, tree density, tree diversity), crop structure and crop conditions (primary indicators: crop type, crop rotation, crop density, crop colour), tillage (primary indicators: type of tillage, timing of tillage), the use of fertilizers (chemical versus organic) and pesticide application.

#### 2.1 Tree cover

Tree cover is defined as the canopy of trees, measured in the field, and recorded as percentage classes of tree cover in three height classes (trees >15 m, 10–15 m, <10 m). Thus, this variable characterized one aspect of agroecosystem management: the farmers’ decision to maintain the canopy of the trees in his or her agroecosystem.

#### 2.2 Tree density

Tree density is defined as the number of trees per area. To measure this, we distinguished three categories of tree density: high density (abundant), medium density, and low density (isolated or no trees). A high number of trees per area guarantee carbon sequestration [35] while a stable microclimate is maintained. This variable, measured in the field, is one the variables that characterize the effect of trees in the agroecosystem.

#### 2.3 Tree diversity

This variable was measured in the field by counting the number of trees species within the plot. In agroecosystems, biodiversity may; (i) contribute to constant biomass production and reduce the risk of crop failure in unpredictable environments, (ii) restore disturbed ecosystem services such as water and nutrient cycling, and (iii) reduce risks of pests and diseases through enhanced biological control or direct pest control [36], [20], [22].

#### 2.4 Crop type

This variable indicates whether the crop is annual, seasonal, or perennial. We obtained this information by observing the type of crop. Annual crops in general have higher environmental impacts, ie: greenhouse emissions, and nutrient leaching, than perennial crops [37].

#### 2.5 Crop rotation

In sustainable farm systems leguminous crops are increasingly used in crop rotations as a source of nutrients, particularly nitrogen for crop growth [38], [39], nitrogen-fixing legumes, contribute to maintaining biodiversity above and in the soil, contribute nitrogen to the soil/plant system, and help avoid the build-up of pest populations [34]. In this study, we asked farmers whether they planted another crop before planting the main crop and whether they practice crop rotation, as crop rotation may assist with weed and pest control [40], [34]. According to Bellon [41], an activity which leads into the maintenance or increase of renewable resources in agroecosystems, is considered as an ecological technology. This variable helped us to characterize the technology used in the agroecosystem.

#### 2.6 Crop density

Crop density is defined as the number of plants (individuals) per area. Three categories were recorded in the field: abundant (high density: 3,000 plants/ha), medium density (1000–1600 plants/ha), and sparse (<1000 plants/ha). This variable also indicated the level of technology applied to the crop, as less intensive techniques typically yield lower densities [42].

#### 2.7 Crop colour

The colour of a crop indicates the nutritional status of the plants; green plants generally have sufficient nutrients, while yellow plants lack nitrogen [43].

#### 2.8 Type of tillage

Type of tillage was categorized into no tillage, manual tillage, and mechanical tillage using machinery, the latter of which generally indicates high disturbance of the soil surface and rapid loss of soil organic carbon and other nutrients [44]. In the field, we asked the farmers how they prepared the land for crop planting.

#### 2.9 Frequency of tillage

This variable indicates frequency of tillage - every year or every 2 years. With this information, it was possible to estimate the frequency of soil disturbance due to tillage.

#### 2.10 Chemical input

Searching for sustainable productions, it is recommended no or low or use of inorganic fertilizers and pesticides [45], [46]. Long term use of some pesticides, as glifosate can decrease earthworm species number, density and biomass [47], [48]. This information allowed us to estimate the amount of chemical fertilizers and pesticides applied within a given area. These variables (chemical fertilizers and pesticides) are included in the indicator for chemical disturbance.

#### 2.11 Green manure

Is defined as the presence or absence of leguminous crops mixed with the principal crop, generally used to increase total soil nitrogen content. Green manures should always be intercropped, as it has been proven that growing legumes with cereal crops decreases N_2_0 emissions [49], and therefore is a sustainable, environmentally friendly practice. Examples of green manure use have been observed in traditional Mesoamerican cultures, for example, intercropping beans, as well as other edible plants, within the *milpa* or traditional maize cropping system [50].

### 3. Index development

Primary indicators were hierarchically aggregated into higher levels, forming intermediate variables, which in turn are structured into a single index that evaluates the ecological condition of a given plot on a scale from 0 (worst) to 1 (best). An index close to zero either would mean that a more environmentally friendly farming techniques need to be implemented or that the plot should be subjected to a fundamental change in land-use, e.g., reforestation, in order to return to a more ecologically sound state. An index close to one, on the other hand, would indicate an ecologically sound land-use. Methods applied in indicator aggregation were (i) simple mathematical operations, ii) sets of if-then rules, and (iii) sets of if -then rules combined with fuzzy logic.

#### 3.1. Aggregation through mathematical operations

Mathematical operations include calculating averages, weighed averages, minimum values, and maximum values. For example, if primary indicator *A* has the value *a* and primary indicator *B* has the value *b*, then the value *x* of the intermediate variable *X* is determined as *x* = (*a*+*b*)/2, *x* = (*w*_1_*a+*w*_2_*b), where *w*1 and *w*2 are weights, or *x* = min(*a*, *b*) or max(*a*, *b*).

#### 3.2. Aggregation by IF-THEN rules

If primary indicator *A* can have the discrete values *a_1_*, *a*_2,_ and *a*_3_, and primary indicator *B* can have the discrete values *b*_1_, *b*_2,_ and *b*_3_, then the value *x* of the intermediate variable *X* is determined by a set of nine (number of levels of *A* x number of levels of *B*) rules. For example, IF *A* = *a*_1_ and *B* = *b*_1_ THEN *X* = *x*_1_.

#### 3.3. Aggregation by IF-THEN rules and fuzzy logic

We used small fuzzy rule-based models for aggregation in the case of non-linear interactions among indicators using a continuous numerical scale or an ordinal scale with a large number of possible values. In classic set theory, an object can either be a member (membership = 1) or not (membership = 0) of a given set. The central idea of fuzzy set theory is that an object may have a partial membership of a set, which consequently may possess all possible values between 0 and 1. The closer an element is to 1, the more it belongs to the set; the closer the element is to 0, the less it belongs to the set. To apply the fuzzy set theory, three steps are involved in calculating the model's output. First, for any observed value of the primary indicators, its corresponding membership value in the fuzzy set domain is calculated (*fuzzification*); second, the memberships of the intermediate variable *X* are calculated, applying the rules in the fuzzy set theory (*fuzzy inference*); third, the fuzzy results are converted into a discrete numerical output (*defuzzification*; see Wieland 2008 for an introduction to fuzzy models). Fuzzy rule-based models have become popular in ecological modelling [51], [52], and several examples exist of its usefulness in the context of ecosystem evaluation, bioindication, and sustainable management [15], [53], [54]. Here, if both primary indicators *A* and *B* can have numerical values from 0 to 1, then the value *x* of the intermediate variable *X* is determined by a series of fuzzy set rules representing the linguistic variables “low *A*”, “medium *A*”, “high *A*”, and “low *B*”, “medium *B*”, and “high *B*”, as well as the output “low *X*”, “medium *X*”, and “high *X*”. The value of the intermediate variable *X* is determined by nine levels of *A* x the number of levels of *B*; for example: If A = low and B = low Then X = [low, medium, or high].

To maintain the number of rules as well as their complexity as low as possible, we aggregated only two variables at a time. A simple example shows the reasoning behind this decision; if there are three primary variables, *A, B,* and *C* with three categories for each variable, a single rule node requires 3*3*3 = 27 If-Then rules of the type “If *A* = *a*_1_, *a*_2_, or *a*_3_ and If *B* = *b*_1_, *b*_2,_ or *b*_3_ and if *C* = *c*_1_, *c*_2,_ or *c*_3_ Then…”, whereas if an additional intermediate variable *X* is introduced to the model, only 18 rules are needed: 3*3 = 9 rules to aggregate *A* and *B* to *X* (If *A* = *a*_1_, *a*_2,_ or *a*_3_ and If *B* = *b*_1_, *b*_2,_ or *b*_3_), and 3*3 = 9 rules to aggregate *X* and *C* (If *X* = *x*_1_, *x*_2,_ or *x*_3_ and If *C* = *c*_1_, *c*_2,_ or *c*_3_).

### 4. Study area and application of the index

**Figure 1:**
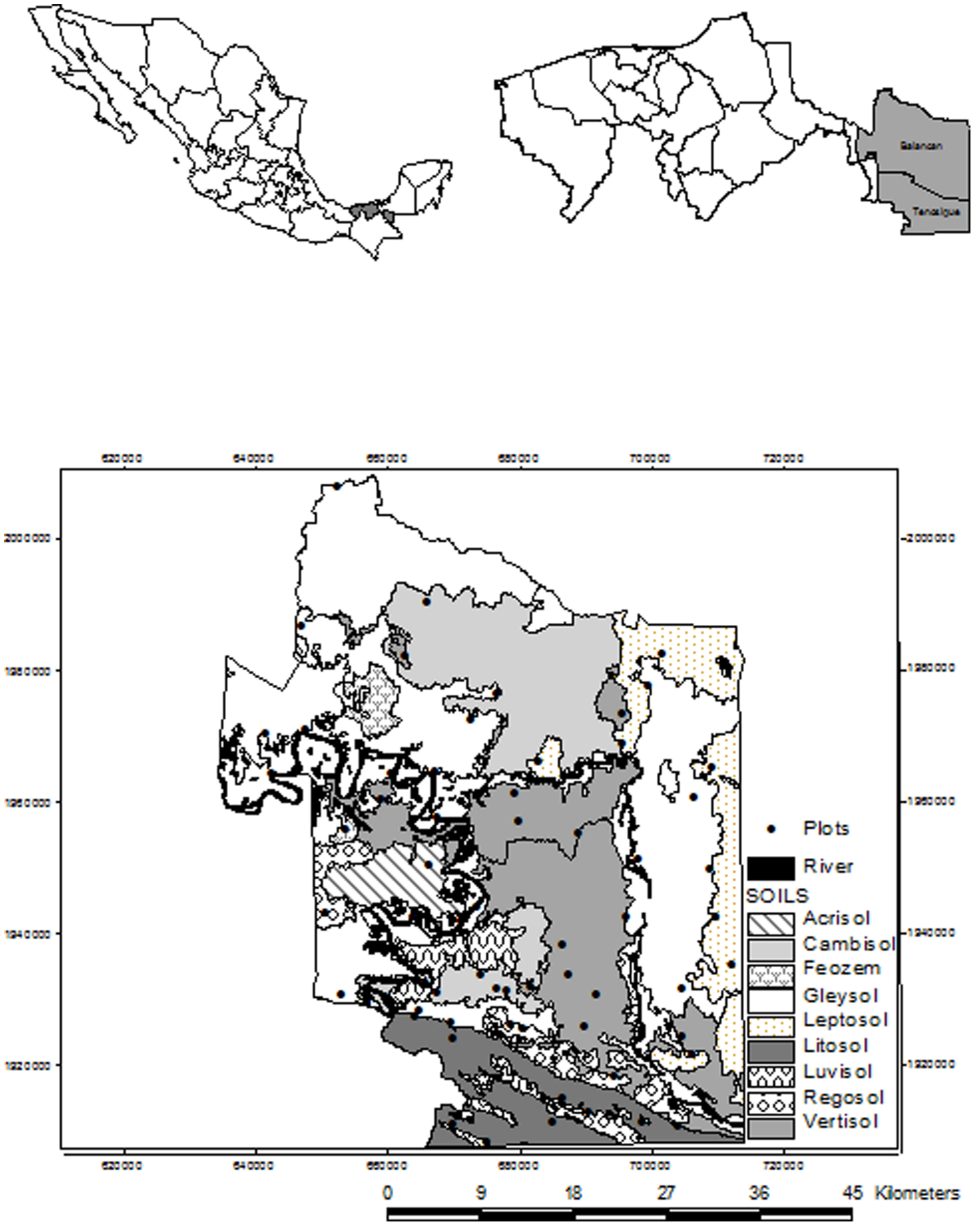
Distribution of 52 evaluated agricultural plots in tropical South-East Mexico.

The state of Tabasco in south-eastern Mexico is characterized by a humid tropical climate with a mean annual rainfall between 1200 and 4000 mm and a mean annual temperature of 27°C [55]. Predominant soils are Gleysols and Fluvisols over alluvial sediments in the plains, Vertisols, Cambisols, Luvisols, and Acrisols over Miocene or Oligocene sediments, and Leptosols and Regosols over limestone mountains [9], [56]. We chose the municipalities Balancán and Tenosique in western Tabasco (17°81′50′′–18°81′00′′ N, 91°80′10′′–91°84′60′′ W) as a study area (Figure 1), we worked with private, *ejidal* (multipurpose land, where owners can or cannot sell the property according to their legal status in the National Agrarian File), and communal lands (coordinates of each plot are shown in Table 1). The region is mainly a plain towards the North (67% of area has an elevation <20 m. a. s. l.) with hills (29%, 20–200 m. a. s. l.), and mountains (4%, max. 640 m. a. s. l.) in the South, comprising a total area of 5474 km^2^. These municipalities have undergone a high degree of land use change over the past 40 years. Until the early 1970 s, this region was still covered by lowland rain forest. The principal form of land use is pastureland, and covers 60% of the land [57]. An additional 30% is cropland, mainly cultivated under small-medium holder systems with seasonal conventional agriculture using high levels of agrochemical inputs (Table 2 & 3). Common crops are maize (*Zea mays*), a variety of hot peppers (*Capsicum* sp), cucumbers (*Cucurbita argyrosperma*), watermelon (*Citrullus lanatus*), perennial fruit crops such as papaya (*Carica papaya*), and biannual crops such as sugar cane (*Saccharum officinarum*) [58].

**Table 1:** Characterization of plots.

Plot #	Municip	Lat	Long	Altitud (mls)	Plot size (ha)	Type of property	Original-vegetation
1	Bal	2007880	652445	43	18	Ejidal	A
2	Bal	1964368	642505	10	0.5	Private	TRF
3	Bal	1970936	647701	11	160	Private	TRF
4	Bal	1957459	667496	14	2	Ejidal	TRF
5	Bal	1964107	660656	14	7	Private	PT
6	Bal	1960297	658989	22	15	Private	TRF
7	Bal	1976300	676424	40	30	Private	TRF
8	Bal	1990326	665947	40	28	Private	TRF
9	Bal	1964590	667264	11	3	Private	RV
10	Bal	1972260	672382	28	2.5	Ejidal	TRF
11	Bal	1950339	666409	49	40	Private	TRF
12	Bal	1966112	682994	40	80	Private	PT
13	Ten	1931754	704474	65	14	Private	TRF
14	Ten	1912951	690381	130	2	Communal	TRF
15	Ten	1932644	665521	15	3	Ejidal	TRF
16	Ten	1926163	678608	56	68	Private	TRF
17	Ten	1933663	674227	59	35	Private	A
18	Ten	1926570	669486	35	8.5	Ejidal	TRF
19	Ten	1942431	663885	21	1.5	Ejidal	TRF
20	Ten	1942068	671156	31	7.25	Ejidal	TRF
21	Ten	1943167	650736	82	14	Ejidal	A
22	Ten	1923838	669841	118	0. 25	Private	TRF
23	Bal	1973301	695548	105	40	Ejidal	TRF
24	Bal	1982392	701383	74	20	Ejidal	A
25	Bal	1965354	709053	44	11	Ejidal	A
26	Bal	1960496	706464	51	44	Ejidal	TRF
27	Bal	1942484	709731	66	10	Ejidal	A
28	Ten	1944148	673673	16	320	Ejidal	TRF
29	Ten	1930350	659650	29	20	Private	TRF
30	Ten	1924549	656910	41	53	Ejidal	TRF
31	Ten	1926226	652978	65	3	Ejidal	TRF
32	Bal	1955194	688843	45	44	Private	TRF
33	Bal	1960980	679265	49	29	Ejidal	TRF
34	Bal	1942552	696222	48	10	Private	TRF
35	Ten	1938120	686470	49	5	Private	TRF
36	Bal	1968750	695595	60	2	Private	A
37	Bal	1977546	699417	53	200	Ejidal	TRF
38	Bal	1949770	708854	53	23	Ejidal	TRF
39	Bal	1951216	697890	46	2	Ejidal	TRF
40	Ten	1931041	667544	31	17	Private	TRF
41	Ten	1927447	664223	3	3	Ejidal	TRF
42	Ten	1928226	664551	31	3	Ejidal	TRF
43	Ten	1930579	691785	54	15	Communal	TRF
44	Ten	1931219	677895	59	63	Private	TRF
45	Ten	1931525	676500	60	6.75	Ejidal	TRF
46	Ten	1908445	675040	140	40	Ejidal	TRF
47	Ten	1910875	669750	222	45	Ejidal	TRF
48	Ten	1924222	704702	42	30	Ejidal	A
49	Ten	1935076	712227	88	20	Ejidal	TRF
50	Ten	1914876	686285	113	4	Ejidal	TRF
51	Ten	1911365	684878	389	22	Ejidal	TRF
52	Ten	1911503	698606	139	10	Ejidal	TRF

Municip: municipality, Bal: Balancan, Ten: Tenosique. TRF: tropical rain forest, PA: pasture, A: acahual (secondary vegetation). Ejidal: multipurpose land, where owners can or cannot sell the property according to their legal status in the National Agrarian File.

**Table 2:** Crop characterization.

Plot	Cycle	Main seasonal orperennial crop	Trees
1	s-s	*Zm*, *Pv*, *Cs*, *Cl*	*Tr*, *Pa*, *Cpa*
2	s-s	*Zm*, *Pv*, *Cs*, *Cl*	*Sh*, *Dg*
3	w-s	*Sv*	*Sa*
4	a-w	*Zm*, *Pv*, *Cpa*	*0*
5	Y	*Zm*, *Pv*, *Cl*, *Cm*	*0*
6	Y	*Zm*	*Cpa*, *Eg*, *Tr*
7	s-a	Ca	Tr
8	Y	*Cp*	*Cp*
9	Y	*Zm*	*0*
10	Y	*Zm*	*0*
11	w-s	*Zm*, *Pv*, *Cl*	*Sm*, *Cpa*
12	Y	*Co*	*Co*
13	s-s	*Zm*	*0*
14	s-s	*Le*, *Se*	*0*
		*Cpa*, *Zm*, *Pv*	
15	s-s	*Cl*, *Ta*, *Mp*,	*Fruit trees*
16	Y	*Jj*	*Gs*, *Hc*, *Cc*, *Tr*
17	Y	*So*	*0*
18	Y	*So*	*0*
19	Y	*So*	*0*
20	Y	*Zm*	*Mi*
21	s-s	*Zm*	*0*
22	Y	*Csi, Cli, Js*	*Co, Pa, Bc, Cn, Mi*
23	s-s	*Zm*, *Cpa*	*Cpe*
24	s-s	*Zm*	*Cpa*
25	s-s	*Zm*	*0*
26	s-s	*Zm*, *Cpa*	*0*
27	s-s	*Zm*, *Ca*	*Sm*
28	Y	*So*	*0*
29	s-s	*Zm*, *Pv*, *Cs*, *Cl*	*0*
30	Y	*Zm*, *Pv*, *Cs*, *Cl*, *Ta*	*0*
31	Y	*Zm*, *Pv*, *Cs*	*0*
		*Cl*, *Os*, *Le*	
32	s-s	*Zm*, *Pv*, *Cpa*, *Cl*, *Os*	*Tr*
33	Y	*Zm*, *Pv*	*Sm*, *Bg*
34	s-s	*Zm*, *Pv*, *Os*	*Cpa*
			*Tr*
35	Y	*Zm*, *Cl*	*Tr*
36	Y	*Zm*, *Pv*, *Cpa*, *Cl*, *Ca*	*0*
37	s-s	*Zm*, *Pv*, *Ca*	*Sm*, *Pa*, *Sh*
38	Y	*Zm*, *Pv*, *Cpa*, *Cl*, *Ta*	*Co*, *Sh*
39	Y	*Zm*, *Pv*, *Cpa*, *Cl*, *Ca*	
		*Cs*, *Cm*	
40	Y	*So*	*Tr*, *Cpe*
			*Co*, *D a*
41	Y	*So*	*Tr*, *Cpe*
			*Co*, *D a*
42	Y	*So*	*Tr*, *Cpe*
			*Co*, *D a*
43	Y	*So*	*Tr*, *Cpe*
			*Co*, *Da*
44	Y	*So*	*Tr*, *Cpe*
			*Co*, *Da*
45	Y	*So*	*Tr*, *Cpe*
			*Co*, *D a*
46	Y	*Zm*, *Pv*, *Cpa*, *Me*	*Sm*
47	Y	*Zm*, *Pv*, *Me*, *Ib*	*Bc*, *Mi*, *Dg*, *Cs*, *Ll*
48	Y	*Zm*, *Pv*, *Cpa*, *Cl*	*0*
49	Y	*Zm*, *Pv*, *Ca*	*Tr*, *Gs*
50	Y	*Zm*, *Pv*, *Cpa*, *Cl*	*Sm*, *Co*, *Ma*
		*Cs*, *Ib*, *Me*	0
51	Y	*Zm*, *Pv*, *Cpa*, *Cs*	*Fruit trees*
52	Y	*Zm*, *Pv*, *Cpa*, *Cl*, *Cs*	*Co*, *Sh*, *Bc*
		*Mp*, *Ca*, *Me*	0

**Cycle**: s–s: spring-summer, w-s: winter-summer, s-a: summer-autumn, Y: all the year. **Main seasonal or perennial crop**: Ca: *Cucurbita argyrosperma* (cushaw pumpkin); Cp: *Carica papaya* (papaya); Cl: *Citrullus lanatus* (watermelon); Cli: *Citrus limon* (lemon); Cm: *Cucumis melo* (muskmelon); Cpa: *Cucurbita pepo* (squash); Cs: *Capsicum* sp. (pepper); Csi: *Citrus sinensis* (orange tree); Ib: *Ipomoea batatas* (sweet potato); Jj: *Jarthropha jurcas* (oil palm); Js: *Jobo spondia* (plum); Le: *Lycopersicon esculentum* (tomato); Me: *Manihot esculenta* (cassava); Mp: *Musa paradisiaca* (banana); Os: *Oryza sativa* (rice); Pv: *Phaseolus vulgaris* (bean), So: *Saccharum officinarum* (sugarcane); Se: *Sechium edule* (pear squash); Sv: *Sorghum vulgare* (milo); Ta: *Triticum aestivum* (wheat ); Zm: *Zea mays* (maize). **Trees**: *Bc: Byrsonima crassifolia* (nanche); *Cc: Crescentia cujete* (calabash tree); *Co: Cedrela odorata* (Mexican cedar); *Cpa: Carludovica palmate* (toquilla palm); *Cpe: Ceiba pentandra* (kapok); Cn: *Cocos nucifera* (Coconut); *Dg: Dialium guianense* (wild tamarind); Eg: *Eucalyptus grandis* (Eucalyptus); *Gs: Gliricidia sepium* (Cocoite, gliricidia, cacao de nance); *Ll: Leucaena leucocephala* (white lead tree, jumbay); *Ma: Mammea americana* (mamey apple); *Mi: Mangifera indica* (mango); *Pa: Persea americana* (avocado); *Sa: Sterculia apetala* (camoruco, manduvi tree); *Sh: Swietenia humilis* (small mahagoni); *Sm: Spondias mombin* (Yellow plum, bai makok); *Tr: Tabebuia rosea* (savannah oak, macuilis).

**Table 3:** Crop land preparation and inputs characterization.

Plot	Tillage	ChemicalFertilization	PesticidesUse	Gm	Oa
1	Ma & Me	NPK 17∶17∶17 & U	Endosulfan**	0	0
2	Ma	0	Chlorpyrifos*	0	0
3	Me	Pholiar	0	0	c s m
4	Ma	0	0	0	0
5	Ma & Me	NPK 17∶17∶17	Chlorpyrifos**	Y	Y
6	Ma & Me	NPK 17∶17∶17	Chlorpyrifos*	0	0
7	Me	0	ID	0	0
8	Me	ID	ID	Y	0
9	Ma & Me	0	Carbofuran*	0	0
10	Me	NPK 18-46-0	ID	0	0
11	Me	NPK 18- 46- 0, U & P	ID	Y	0
12	Me	NPK 18-46-0	ID	0	0
13	Me	NPK 17∶17∶17 & U	Chlorothalonil**	0	0
			Chlorpyrifos*		
14	Ma	0	Endosulfan*	0	0
15	Me	0	0	Y	0
16	Ma	Urea	0	0	0
17	Me	NPK 19-19-19	ID	0	0
18	Me	NPK 19-19-19	ID	0	0
19	Me	Urea	ID	0	bg
20	Me	0	Methilic*	0	0
21	Me	NPK 17∶17∶17 & U	Zeta**	0	0
22	Ma	0	0	0	0
23	Me	NPK 17∶17∶17 & U	Chlorpyrifos**	0	lcf
24	Me	Urea & P	Zeta*	Y	lcf
25	Me	Urea	(2,4-D AMINA)*	Y	0
26	Me	NPK 17∶17∶17 & U	(Z)-(1R,3R)**	0	0
27	Me	0	Zeta*	N	0
28	Me	NPK 17∶17∶17	(RS)*	0	0
29	Me	NPK 17∶17∶17 & U	Chlorpyrifos**	0	0
30	Ma	0	Chlorpyrifos**	Y	0
31	Me	NPK 17∶17∶17 & U	Chlorpyrifos*	0	0
32	Me	0	0	Y	0
33	Me	0	0	0	0
34	Me	0	0	0	0
35	Me	NPK 17∶17∶17 & U	0	0	0
36	Me	NPK 17∶17∶17 & U	Chlorpyrifos*	Y	0
37	Me	0	Urea	0	0
38	Me	NPK 17∶17∶17 & U	Carbofuran*	Y	0
39	Me	NPK 17∶17∶17 & U	Chlorpyrifos**	0	0
40	Me	NPK 17∶17∶17	(Z)-(1R,3R)*	0	0
41	Me	NPK 17∶17∶17 & U	(Z)-(1R,3R)*	0	0
42	Me	NPK 17∶17∶17 & U	(Z)-(1R,3R)*	0	0
43	Me	NPK 17∶17∶17 & U	(Z)-(1R,3R)*	0	0
44	Me	NPK 17∶17∶17 & U	(Z)-(1R,3R)*	0	0
45	Me	NPK 17∶17∶17 & U	(Z)-(1R,3R)*	0	0
46	Ma	0	0	Y	0
47	Me	U	0	0	0
48	Ma &Sb	Urea	Chlorpyrifos**	0	0
49	Me	Urea & P	Zeta*	0	0
50	Ma	Urea	Endosulfan*	0	0
51	Ma	0	ID	0	0
52	Me	NPK 17∶17∶17 & U	Chlorpyrifos*	0	0

**Tillage**: Ma: Manual; Me: Mechanized. **Pesticides**: Endosulfan: 6,7,8,9,10,10-Hexachlor- 1,5,5a,6,9,9a-Hexahidro-6,9-metane-2,4,3-benzodioxatiepin-3-oxide; Chlorpyrifos: 0,0-dimetil 0-(3,5,6-trichlore-2-piridinil) fosforotioate (33.8%), Permetrine: 3-fenoxibenzil (1RS)-cis, trans-3-(2,2 diclorovinil)-2,2 dimetil ciclopropane-carboxilate (4.8%); Carbofuran: 2,3 Dihidro-2,2-dimetil-7-benzofuranil metil carbamate; Chlorothalonil: Tetrachloroisoftalonitrile; Zeta: Zeta-cipermetrine a-ciano-3-(fenoxifenil) metil (±) cis-trans; (Z)-(1R,3R): (Z)-(1R,3R)-3-(2-chloro-3,3,3-trifluoroprop-1-enil)-2,2-dimetilcichlopropanecarboxilate de (S)-α-ciano-3-fenoxibencile & (Z)-(1S,3S)-3-(2-chloro-3,3,3-trifluoroprop-1-enil)-2,2-dimetilcichlopropanecarboxilate de (R)-α-ciano-3-fenoxibencile; (RS): (RS)- alfa- ciano-3-fenoxybencil(1RS)-cis-trans-3-(2,2-dichlorvinil)- 1,1-dimetilcichlopropanecasrboxilate. * Once per year, ** 2 per year. **Chemical Fertilization**: U: Urea, P: Phosphorus. **Gm**: Green manure, Y: Yes, 0: null application. **Oa**: Organic amendments: csm: cow, sheep manure, bg: burned grass, lcf: last crop fallow, 0: null application.

We chose 52 farms in the study area (Table 1), and selected those farms whose main economic activity (100%) is agriculture and chose one agricultural plot from each farm for evaluation. There were annual and biannual crops with or without trees (Table 2). Average plot size was 32.4±55.1 ha, and the average time that the plot had been used for a given crop system was 2.5±3.0 years.

We questioned farmers as to frequency, amount and type of chemical fertilizers and pesticides applied per area of cropland. After their verbal consent, farmers gave us their complete name and signed next to their name on the record sheet, validating all the information. Due to the fact that we only asked about the use of fertilizers and management of the land, the procedure approval from the Ethics Committee was not required.

Between March and October 2004, for each plot, the values of the primary indicators were determined and the index of ecological condition was calculated. Prior to index calculation, the plots were also evaluated by experts (2 scientists, each with over 15 years of experience in agroecology) on a scale from 0 (poor condition) to 1 (good condition) in order to test the correlation between the experts’ opinions and the index.

Data was normalized for carrying out multiple regression with plot size, altitude of plot site, or previous vegetation cover and the ecological index.

Finally we located the index in this public web address: http://201.116.78.102/~modelo/Index.html, where farmers in the near future can introduce independent data sets and evaluate or monitor their own agro ecosystems.

## Results

### 1. Index structure

**Figure 2:**
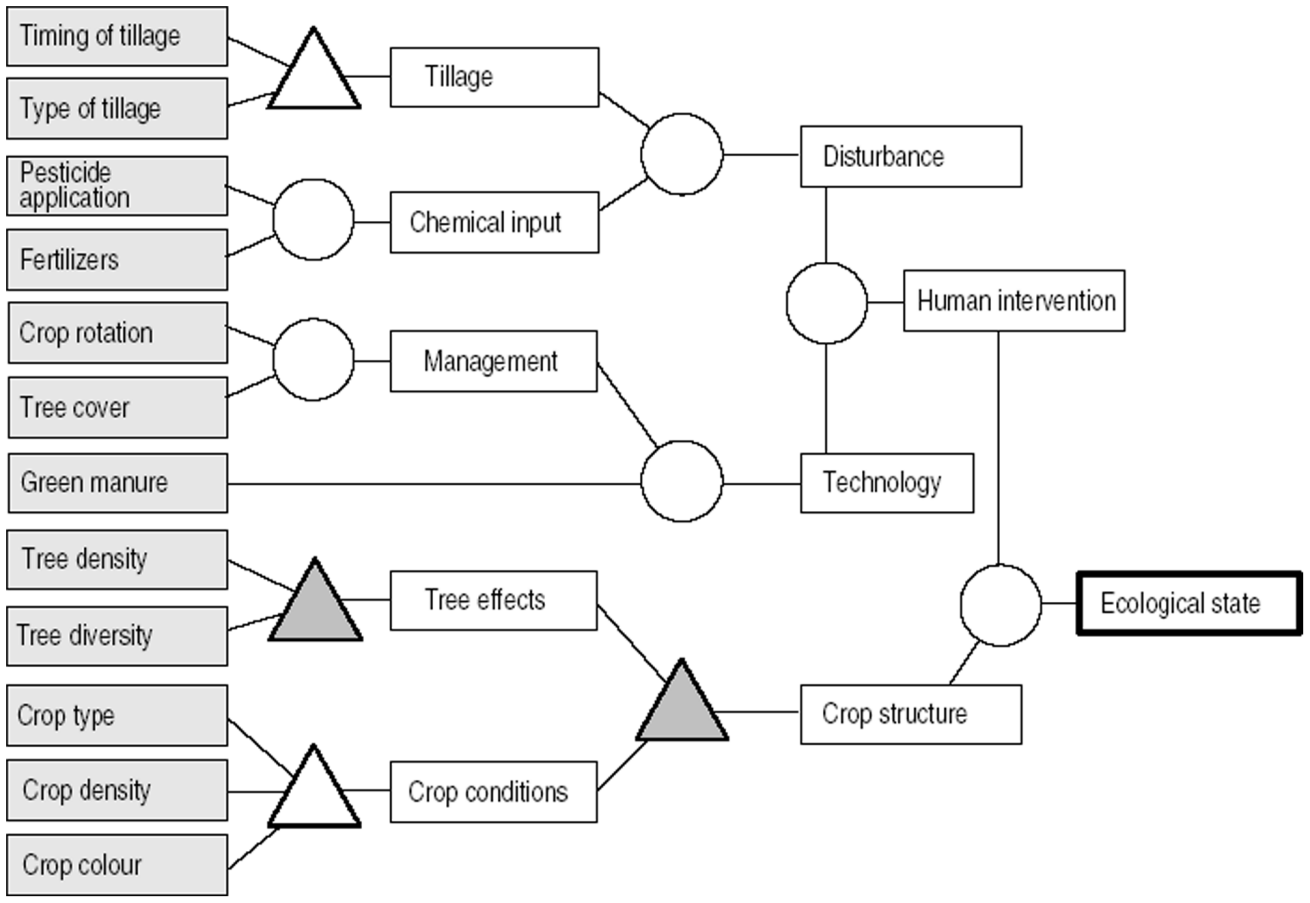
Structure of the Index of ecological condition of tropical agroecosystems. Primary indicators are shaded in grey. Circles represent simple mathematic algorithms, white triangles represent rule sets, and grey triangles represent rule sets, and grey triangles represent rule sets based on fuzzy logic. This index was presented together with other indexes within a frame of Indicators of environmentally sound land use in the humid tropics [15].

Ten nodes were used to aggregate primary indicators to the final index of ecological conditions of agricultural systems (Figure 2). Six of these used simple mathematical operations; two were based on rule sets, and two on fuzzy rule-based models (Table 4).

**Table 4:** Description of the measurement levels of the variables.

			Best	Intermediate			Worst
	Groupal variables	Field data variables		5 levels			
			1	0.75	0.5	0.25	0
	Management	Tree cover	all year	presence polyculture	present	rare	null
	Tree effect	Tree density	high	medium	low	isolated	null
				4 levels			
			1	0.66	0.33		0
		Tree diversity	>4 species	4 species	2 species		1 species
Crop structure	Crop conditions	Crop type	perennial polyculture	perennial monoculture	biannual		annual
				3 levels			
			1	0.33			0
	Plough (tillage)	Tillage time	null	frequent			constant
		Tillage form	null	manual			technical
							
Disturbance	Chemical inputs	Pesticides	null	frequent			constant
		Fertilizers	null	frequent			constant
	Management	Crop rotation	constant	frequent			null
Technology		Green manure	constant	frequent			null
				2 levels			
			1				0
	Crop conditions	Crop density	abundant				disperse
		Crop coleur	green				yellow

### 2. Index application

#### 2.1. General characterization of sampled plots

A total of 67% of the plots were cultivated after cutting of primary lowland forest, 24% after cutting secondary forests of various ages, and 1.5% after cutting riparian vegetation, the rest conversion from pastureland (livestock production) to crop system. On 63% of the farms, maize, beans and pumpkins were cultivated, 13% sugar cane, 12% watermelon, 9% rice and 3% pepper. On 68.5% of the plots, trees were scattered among the crops. Conventional tillage was used on 55.5% of the plots, and pesticides were used on 79.4%, green manure was used on 21% of the plots and chemical fertilizers on 67%. All farmers understood the meaning of the variables evaluated for their plots (for a complete list of plots, see Table 5).

**Table 5:** Normalized Ecological Index for plots evaluated (52).

Plot	TT	TF	P	F	GM	RC	CA	DA	DivA	CT	CD	Ccol	Ev	Index
1	n	m	c	c	n	n	>1	l	>4	pm	a	g	0.24	0.56
2	n	n	c	n	n	n	>1	i	>4	a	a	g	0.35	0.5
3	c	m	n	c	c	n	>1	i	>4	a	a	g	0.26	0.56
4	n	n	n	n	n	n	n	n	1	a	a	g	0.01	0.37
5	n	n	c	c	c	c	>1	i	>4	a	a	g	0.09	0.62
6	n	m	c	c	n	n	>1	l	>4	a	a	g	0.22	0.5
7	c	t	c	n	n	n	>1	i	>4	a	d	b	0	0.15
8	c	t	c	c	c	c	n	n	1	pp	d	g	0.1	0.12
9	n	t	c	n	n	n	n	n	1	a	a	g	0.09	0.25
10	c	t	c	c	n	n	n	n	1	a	d	b	0	0
11	c	t	c	c	n	c	>1	i	>4	pm	ID	ID	0.03	0.21
12	c	t	c	c	n	n	>1	h	>4	ba	a	g	0.2	0.5
13	n	t	c	c	n	n	>1	i	>4	a	a	g	0.03	0.5
14	n	n	c	n	n	n	n	n	1	pp	a	g	0.29	0.37
15	n	t	n	n	n	c	>1	h	>4	pp	a	g	0.2	0.81
16	n	n	c	c	n	n	>1	l	>4	ba	a	g	0.25	0.5
17	c	t	c	c	n	n	n	n	1	a	a	g	0	0.25
18	c	t	c	c	n	n	n	n	1	a	d	g	0	0
19	c	t	c	c	c	n	n	n	1	a	d	g	0	0.06
20	n	t	c	n	n	n	>1	i	>4	a	a	g	0.1	0.5
21	c	t	c	c	n	n	n	n	1	a	a	g	0	0.25
22	n	n	n	n	n	n	>1	i	>4	pp	d	g	0.33	0.62
23	n	t	c	c	c	n	>1	i	>4	a	a	g	0.15	0.56
24	n	t	c	c	c	c	>1	l	>4	a	a	g	0.2	0.62
25	n	n	c	c	n	c	n	n	1	a	d	g	0.01	0.06
26	n	m	c	c	n	n	n	n	1	a	a	g	0.25	0.25
27	n	t	c	n	n	n	>1	i	>4	a	a	g	0.01	0.5
28	n	t	c	c	n	n	n	n	1	a	a	g	0	0.25
29	c	t	c	c	n	n	n	n	1	pm	a	g	0.01	0.37
30	n	m	c	n	n	c	n	n	1	pm	a	g	0.1	0.43
31	c	t	c	c	n	n	n	n	1	a	a	g	0.01	0.25
32	c	t	n	n	n	c	>1	i	>4	a	d	y	0.09	0.34
33	c	t	n	n	n	n	>1	h	>4	a	a	g	0.15	0.62
34	c	t	n	n	n	n	>1	i	>4	ba	a	g	0.13	0.62
35	c	t	n	c	n	n	>1	l	>4	a	a	g	0.01	0.5
36	c	t	c	c	n	c	n	n	1	a	a	g	0	0.31
37	c	t	c	n	n	n	>1	i	>4	pm	a	y	0.01	0.15
38	c	t	c	c	n	c	>1	h	>4	pm	a	g	0.2	0.68
39	c	t	c	c	n	n	>1	l	>4	a	a	b	0.06	0.18
40	c	t	c	c	n	n	>1	l	>4	a	a	g	0.19	0.5
41	c	t	c	c	n	n	>1	l	>4	a	a	g	0.19	0.5
42	c	t	c	c	n	n	>1	l	>4	a	a	g	0.17	0.5
43	c	t	c	c	n	n	>1	l	>4	a	a	g	0.2	0.5
44	c	t	c	c	n	n	>1	l	>4	a	a	g	0.2	0.5
45	c	t	c	c	n	n	>1	l	>4	a	a	g	0.2	0.5
46	n	n	n	n	n	c	>1	l	>4	pp	a	b	0.6	0.37
47	n	t	n	c	n	n	>1	l	>4	pp	d	g	0.18	0.5
48	c	n	c	c	n	n	n	n	1	a	a	b	0	0
49	c	t	c	c	n	n	>1	l	>4	a	a	b	0.07	0.18
50	c	n	c	c	n	n	>1	l	>4	pm	a	g	0.2	0.56
51	c	n	c	n	n	n	>1	l	>4	pm	a	g	0.15	0.56
52	c	t	c	c	n	n	>1	l	>4	pm	a	ID	0.24	0.18

TT: Tillage Time: c: >once per year; n: once per year; TF: Tillage Form: n: null, m: manual, t: technical; P. Pesticide use: c: constant, n: null, F: Use of chemical Fertilizers: c: constant, n: null; GM: Green Manure, c: constant, n: null; RC: Crop Rotation: c: constant, n: null; CA: tree cover: >1 year, n: null; DA: tree density: h: high, l: low, i: isolated, n:null; DivA: tree diversity: >4 species, 1 species, CT: crop type: a: annual, ba: biannual, pp: perennial polyculture, pm: perennial monoculture; CD: crop density: a: abundant, d: disperse; Ccol: crop colour: g: green, b: brown, y: yellow; EV: evaluation.

#### 2.2. Plot evaluation with the index

**Figure 3:**
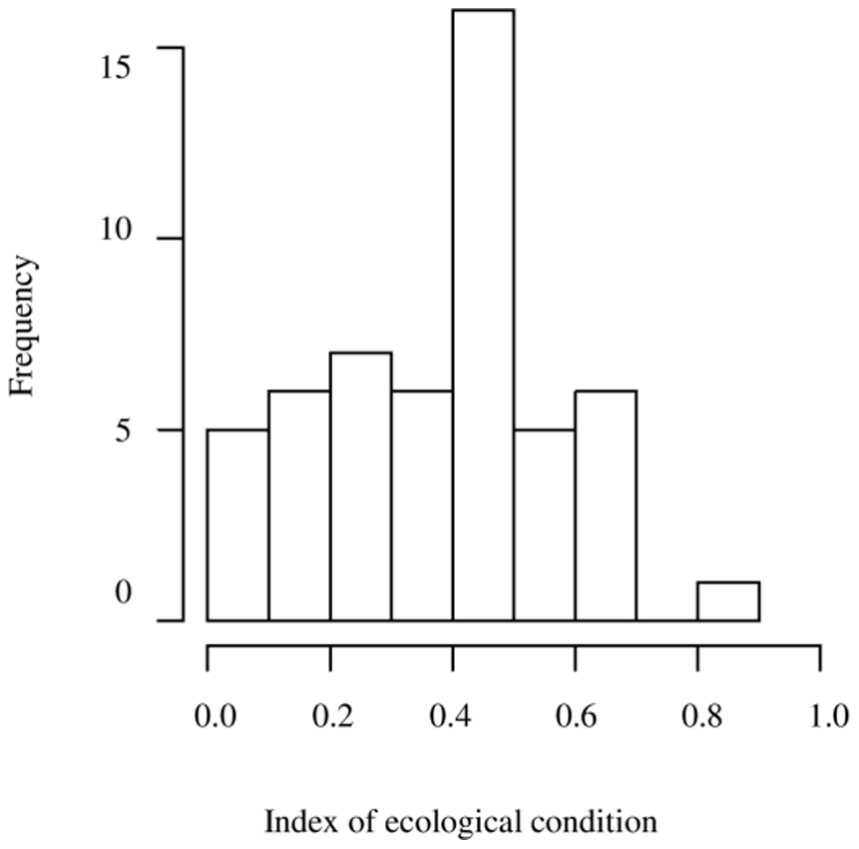
Frequency histogram of values of the index of ecological condition applied to 52 plots in South-East Mexico.

Ecological condition of the plots ranges from 0.0 to 0.8125 (Figure 3; Table 5). One plot which was intercropped with timber and fruit trees presented the highest value. This site was characterized by an absence of agrochemical use, use of green manure, presence of annual crop rotation, and high tree diversity. We found 18 plots with index values of 0–0.25, 7 plots with values of 0.3–0.5, and 26 plots with values of 0.5–0.7. Thus, the majority of the plots evaluated in this study had an intermediate index value. 50% of the plots with this intermediate index are *ejidal* property, 38% private land, and 12% is under another type of ownership (smallholder or communal). 80% of these intermediate plots were lowland rain forest before being converted into agricultural land, 12% were secondary vegetation, and 8% were pastureland (Table 1). One might believe that a prior forest condition implies a more environmentally friendly agroecosystem that allows for preserving a more diverse system. However, plots with low index values were also lowland rain forest before being turned into agricultural land (Table 5). In this case, the land managers or owners decided to deforest the land to subsequently plant annual crops. 13 plots were larger than 40 ha (Table 1). All of these were previously covered with lowland rain forest; the ecological index ranges from 0.5–0.56 for 46% of these plots, and another 23% have an ecological index of 0.37–0.43. 10 plots fell under the smallest size category (0.25–3 ha) and had an ecological index of 0.5–0.68 (4 plots), 0 to 0.18 (5 plots), and 0.39 (1 plot).

Carrying out multiple regression with normalized data, we did not observe significant correlations between plot size, altitude of plot site, or previous vegetation cover and the ecological index (Kendall’s Tau, T = 0.016 p = 0.98, T = 0.11 p = 0.90, T = −0.31 p = 0.75, respectively). Therefore, in this study, it seems that neither size nor location of the plot determines the type of plot management; rather, plot management is likely determined by government development programs and traditional farming techniques.

### 3. Correlation between index and expert opinion

**Figure 4:**
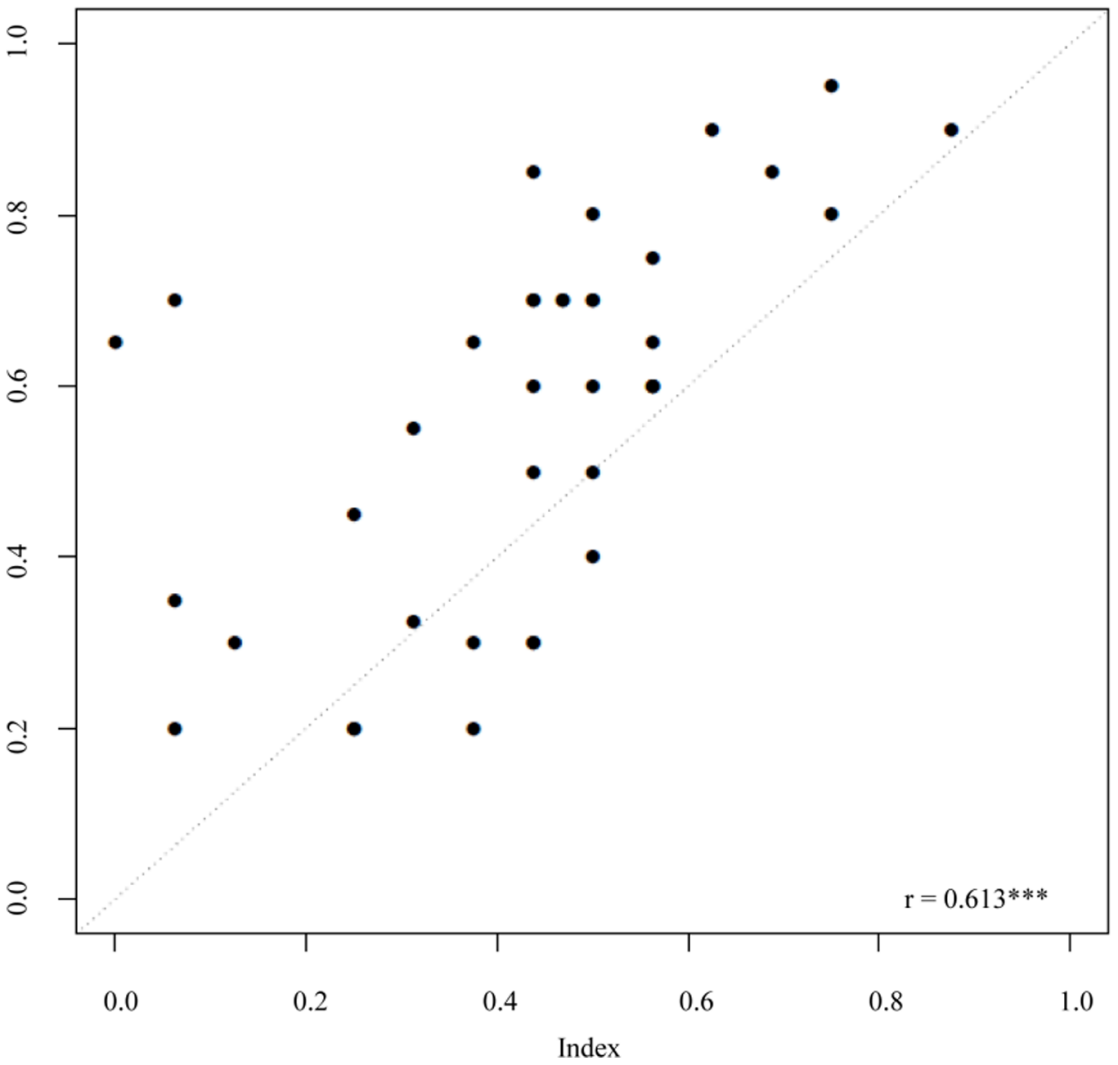
Scatterplot of the values of the index of ecological condition applied to 52 plots in South-East Mexico (x-axis) versus quality values between 0 (worst) and 1 (best) assigned by experts to the same plots (y-axis).

We obtained a Pearson correlation coefficient of *r^2^* = 0.61 (*p* = 2.4e-06, *d.f.* = 49) between the index and the values determined by independent experts, indicating a satisfactory correspondence (Figure 4). However, the experts systematically awarded higher scores to the plots than did the index. Moreover, they suggested to include additional variables to the index which would yield better information regarding (i) type of organic inputs to the crop system, (ii) types of pest and disease control used, (iii) number of native plant species among the crop, (iv) origin of crop seeds, (v) vegetation surrounding the crop, (vi) presence of vertebrate fauna, and (vii) diversity of soil macroinvertebrates.

## Discussion

Since the Rio Earth Summit, there has been a concerted effort to construct indicators to monitor progress toward sustainable development [59]. Most of these indicators have been developed in Europe (10) and Asia (2) [60]. In Latin America, sustainable indicators have been developed by Astier et al. [61]; this index mainly focuses on subsistence level agriculture, and evaluates the sustainability of a system.

Our index is geared toward small and medium scale producers who principally grow for market and have a fairly large crop area (32.4 ha on average). According to Bockstaller and Girardin [62], indicators must be elaborated according to a scientific approach, and one of the important steps in this elaboration is validation.

Our index was developed according to the consensus of a group of scientists, with knowledge in agroecology, and was validated independently by 2 scientists, each with over15 years of experience in agroecology. The evaluation included 3 important steps: design validation, output validation, and end use validation [62].

In previous studies, only seven indicators have been used to evaluate farm systems: crop diversity, crop succession, pesticide use, nitrogen level, phosphorus level, soil organic matter, and irrigation methods [63]. All of these practices depend on the farmer's decision, and to a large extent they impact the environment.

Our index is based on qualitative and quantitative concrete data and includes most of these 7 indicators, except nitrogen and phosphorus, both of which are observed indirectly via plant health, through the crop colour indicator (according to whether plants have a greenish or yellowish colour); organic matter, which is indirectly characterized by the technology applied in the system (green manure indicator, Figure 2); and irrigation, which in this study was not evaluated, given that all plots evaluated were only used for seasonal rainfed agriculture, according to local rainfall patterns.

At the farm level there are indicators that evaluate the environmental impact of the agricultural practices and indicators that evaluate the effect of those practices at the local and global level [60].

Our index includes both types of indicators; evaluating agricultural practices: those variables taken in the field: type and frequency of tillage, pesticide application, fertilizer use, crop rotation, tree cover and density, green manure, crop density (see Table 2 & 3). Effect indicators used were disturbance (mainly soil disturbance) measured by tillage (frequency and type) and chemical inputs, technology used, and crop structure (see index, Figure 2). Some existing indexes focus on evaluation or evolution of environmental performance, thus encouraging environmentally sound practices [60], such as crop rotation, organic fertilizers, and no-tillage. Meanwhile, our index identifies the indicator that has the highest environmental impacts in each plot evaluated, with the idea that the farmer could potentially improve these with a given practice, ie. to monitor the soil ecological condition via the use of a soil macroinvertebrates index [64], where the lack of macroinvertebrates informs of a severe negative activity as pollution or conventional tillage.

In developing the variables to be included in our index, we reviewed bibliographic studies and carried out field work obtaining data which we hoped would reflect the negative and environmentally friendly practices commonly used in agro ecosystems of south-eastern Mexico. The index can be used by farmers, using the following web address: http://201.116.78.102/~modelo/Index.html.

However, we did not evaluate certain indicators such as water use, and water quality, as did - for example - the index (monitoring tool) of ecological indicators used on a Flemish dairy farm [65]. Nor did we evaluate environmental impacts due to energy consumption [66]. Within a tropical framework, in south-eastern Mexico, the priorities were to identify those practices that were soil perturbing and environment polluting, practices that can be modified by the farmers, by an attitude changing. Nambiar et al. [16], proposed an agricultural sustainability index (ASI) to measure sustainability as a function of biophysical, chemical, economic, and social indicators, our index only measures the ecological state of the agroecosystem, and provides easy to use tools for improving those practices which negatively impact the environment. Van der Werf and Petit [60], state that indicators based on farmer practices cost less in data collection but do not allow for an actual evaluation of environmental impact. In the case of our study, the experts’ evaluation correlated satisfactorily with the index, although the index rendered more penalizing scores than did the experts. Some improvements must be made to our index, relating quality and quantity of the applied inputs for instance; the index should specify the kind of manures used and then to evaluate their effect when added to the systems. The consulted experts found important to integrate this information into the index, they also found that the possible relations among different crops and environmental effects of using cow manure, vermicompost, or traditional compost have to be considered. Another variable which the experts suggested should be added to the index is the presence of natural vegetation surrounding the crop. Farmers see advantages of having crops surrounded by secondary forest, diversity in the agricultural area can be increased, ie when different pollinators arrive.

The advantages of using this index is that the common agricultural practices (mechanized land preparation and use of common pesticides ie. Carbofuran, Chlorpyrifos), evaluated in this study as indicators are used throughout the world; over time, through practice, the index may be improved. Farmers and other land owners may realize which of the practices they use are disturbing the environment, due to the fact that these practices generate a value in each of the evaluated indicators. The variables obtained in the field contribute to the information of each of the indicators, and the index is the compendium of all the indicators. Our index doesn’t give a sustainability measure, because it does not include socioeconomic indicators of the farms. Further studies are required in order to observe the acceptance of this index by farmers in a regional scale.

## Supporting Information

Figure S1 Indicators of environmentally sound land use in the humid tropics [15].
(PDF)
