# Supplementary material for: A Multi-Criteria Index for Ecological Evaluation of Tropical Agriculture in Southeastern Mexico
Source: PLoS One. 2014 Nov 18;9(11):e112493. doi: 10.1371/journal.pone.0112493 (PMC4236094; doi:10.1371/journal.pone.0112493)
Supplement: Figure S1 — Indicators of environmentally sound land use in the humid tropics [15]. (PDF) [file pone.0112493.s001.pdf]

Provided for non-commercial research and education use.
Not for reproduction, distribution or commercial use.

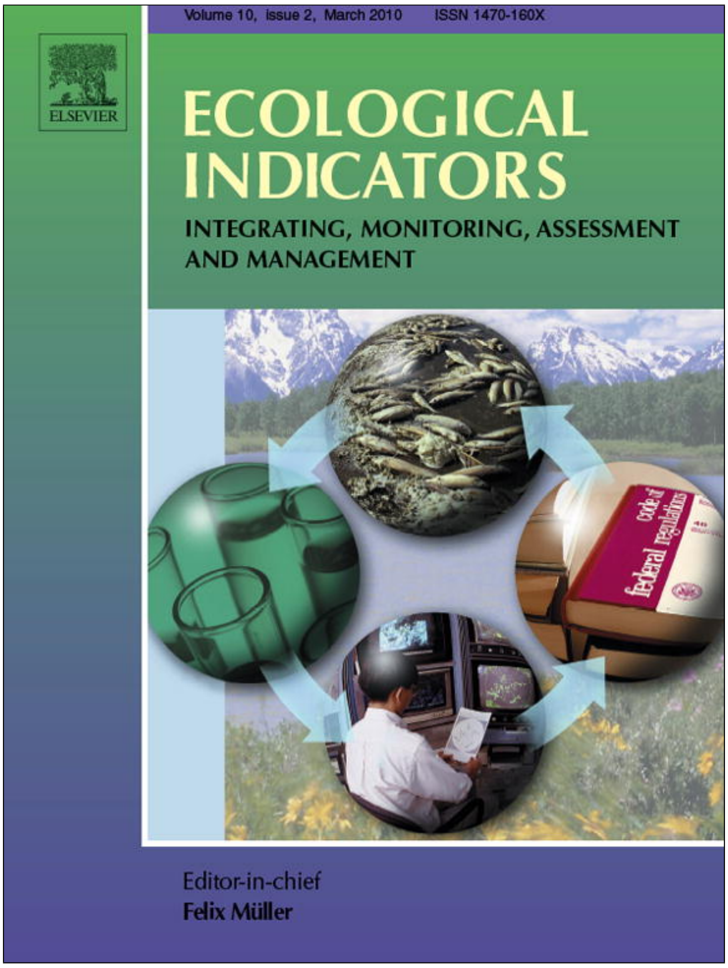


This article appeared in a journal published by Elsevier. The attached copy is furnished to the author for internal non-commercial research and education use, including for instruction at the authors institution and sharing with colleagues.

Other uses, including reproduction and distribution, or selling or licensing copies, or posting to personal, institutional or third party websites are prohibited.

In most cases authors are permitted to post their version of the article (e.g. in Word or Tex form) to their personal website or institutional repository. Authors requiring further information regarding Elsevier's archiving and manuscript policies are encouraged to visit:

http://www.elsevier.com/copyright

Author's personal copy

Ecological Indicators 10 (2010) 320–329

Contents lists available at ScienceDirect

Ecological Indicators

journal homepage: www.elsevier.com/locate/ecolind

# Indicators of environmentally sound land use in the humid tropics: The potential roles of expert opinion, knowledge engineering and knowledge discovery

Christian Kampichler [a,*], Salvador Hernández-Daumás [b], Susana Ochoa-Gaona [b], Violette Geissen [b], Esperanza Huerta [b], Ben de Jong [b]

[a] Universidad Juárez Autónoma de Tabasco, División Académica de Ciencias Biológicas, 86150 Villahermosa, Tabasco, Mexico
[b] El Colegio de la Frontera Sur, Unidad Villahermosa, 86270 Centro, Tabasco, Mexico

ARTICLE INFO

*Article history:*
Received 26 February 2009
Received in revised form 8 June 2009
Accepted 10 June 2009

*Keywords:*
Fuzzy rule-based model
Indicator development
Machine-learning
Regression tree
Sustainable land use
Tropical agriculture
Tropical cattle-farming
Tropical forest
Tropical soils

ABSTRACT

Despite abundant literature on indicators for sustainable resource management, practical tools to help local users to apply its general concepts at a local to regional level are scarce. This means that decisions over land evaluation and land use at a local level are often not based on the formal application of indicators or decision support systems for environmentally sound management but instead on the opinion of local expertise, for instance forest managers, cattle breeders, farmers and/or academics. This is particularly seen to be the case in the tropics where access to modern communication and information technologies is restricted.

As the opinions of experts are often based on and influenced by personal experience, intuition, heuristics and bias, their evaluations and decision are often unclear to the non-expert working at a local level. In order to make their reasoning more comprehensible to the non-expert, the ecological condition of 176 plots in the tropical Southeast of Mexico were evaluated by experts on soil fertility, forest management, cattle breeding and agriculture. With the assistance of a knowledge engineer (one who converts expert knowledge and reasoning into a model), these expert opinions and reasoning were then translated into a formal computer model.

As an alternative approach we applied a knowledge discovery technique, namely the induction of regression trees and automatically developed models using the expert evaluations as training data. Where knowledge engineering was tedious and time consuming, regression models could be rapidly generated. Moreover, the correspondence between regression trees and expert opinions was considerably higher than the correspondence between expert opinion and their own models. The regression trees used less explicative variables than the models generated by the experts. The minimisation of sampling effort due to variable space reduction means that the application of regression tree induction has a high potential for a rapid development of indicators for narrowly defined ecological assessments, needed for decision making on a local or regional scale.

© 2009 Elsevier Ltd. All rights reserved.

## 1. Introduction

Since the report of the World Commission of Environment and Development (1987), better known as the “Brundtland Report”, and the UN Conference on Environment and Development (1992) in Rio de Janeiro, the concept of sustainability has gained worldwide acceptance and popularity. It is generally agreed that sustainability indicators are necessary to put the sustainability concept into effect and to introduce it into government policies. There is an abundance of literature on holistic indicators for sustainable development as well as on indicators for specific dimensions of sustainability, for example, the environmentally sound management of a variety of natural resources such as forestry, agriculture and animal production (the works of Büchs, 2003; Mendoza and Prabhu, 2003; Karlen et al., 2004; Gamborg and Sandøe, 2005; OECD, 2008 are but a few).

However, it is only recently that practical tools which can help local users to apply these general concepts at the local to regional levels have emerged (Hurni, 2000). Land evaluation and land-use decisions on a local scale are instead often based on the opinion of local experts, as opposed to the formal application of indicators or decision support systems for environmentally sound resource management. This is particularly evident in the tropics where, due to restricted access to modern communication and information technologies, local decision makers rely on the expertise of local academics, forest managers, cattle breeders and/or farmers.

* Corresponding author.
*E-mail address:* christian.kampichler@web.de (C. Kampichler).

1470-160X/$ – see front matter © 2009 Elsevier Ltd. All rights reserved.
doi:10.1016/j.ecolind.2009.06.010

Author's personal copy



However, similar problems arise even in developed countries; Sutherland et al. (2004) reported that more than 75% of the knowledge sources consulted for conservation management actions in a UK nature reserve are based on common sense and personal experience, rather than scientific evidence.

If the domain expert's reasoning is to be understood by decision-makers and users it must first be translated into a formal model which will typically, but not necessarily take the form of a computer programme. Two key parts in the development of a formal model are 'knowledge acquisition' (Finlay and Dix, 1996), the gleaning of knowledge from domain experts which will be required by the knowledge engineer in the formation of a formal model and 'knowledge engineering' (Medsker and Liebowitz, 1994), the process of designing, developing, testing and implementing a computer programme for a formal model. The acquisition of knowledge from experts has proved to be a difficult task as many expert system projects have shown, experts are notoriously bad in communicating what they know in a formalised manner. Also, checking the represented experts' knowledge for consistency and completeness, for example as a set of IF-THEN rules, can be a laborious and time consuming task.

The development of a formal model will require several interviews between the knowledge engineer and domain expert in order to fill in any gaps in knowledge, resolve doubts and confirm the model structure. Also, to facilitate clearer communication the expert must become familiar with at least a very basic knowledge of the inference methods applied, fuzzy logic being one example. This can lead to what is referred to in expert systems literature as a 'knowledge acquisition bottleneck' (Medsker and Liebowitz, 1994). To remedy this problem, ways in which expert opinions can be automatically translated into a formal model have been sought, for example, the application of learning algorithms to automatically generate models from training data, a process called machine learning or knowledge discovery. Recently, frameworks have been proposed to aid researchers in designing fuzzy indices of environmental conditions (Marchini et al., 2009); unfortunately these were published too late to be applied in this study.

For the purpose of this study all three strategies were applied, expert opinion for evaluating land use types, knowledge engineering by converting the reasoning of the domain experts into a formalised model and knowledge discovery by automatically inducing a model using the domain expert's opinion as training cases.

First of all, using one domain expert for each land use and soil type, evaluations were made on a number of sites based on a set of primary indicators chosen by each expert. The experts then tried to explicitly represent their reasoning in a hierarchical model that should predict the ecological state of each site. This posed the question of whether the end results would correspond with one another, as a synthetic domain experts' evaluation (based on experience and heuristics) will not necessarily coincide with an analytical explanation, even when both are provided by the same expert.

Finally, to discover how far an automated time saving procedure could replace the difficult time consuming process of knowledge acquisition, the evaluations made by each domain expert were used as training data in the automated induction of regression trees. It was also necessary to find out whether the automatically induced model would contain every primary indicator chosen by the domain expert, or if some of them would become redundant.

The two questions addressed in this paper are: (1) Do the predictions of formal models, based on the knowledge and experience of domain experts, actually correspond to the evaluations made by the same experts? (2) Can time-saving machine learning methods be used to develop evaluation models and, if so, how do they correspond to the models developed by the domain experts?

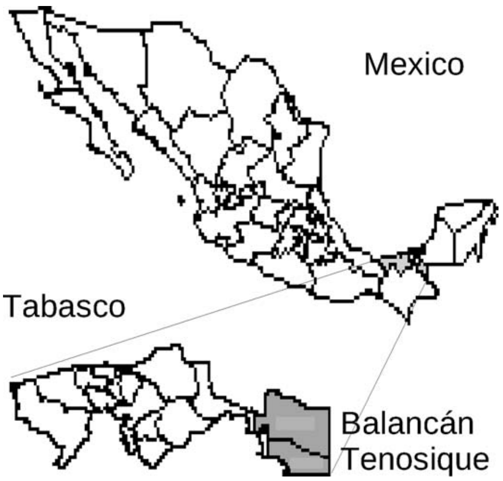


**Fig. 1.** Location of the Mexican State of Tabasco and the municipalities Balancán and Tenosique.

## 2. Materials and methods

We evaluated the ecological state of forested sites, cattle-breeding ranches and agricultural areas, as well as the soils in the State of Tabasco, SE Mexico, a region which has undergone drastic land use changes in the last few decades, such as the conversion of tropical forest to cattle-breeding areas (Challenger, 1998). We randomly chose 176 sites distributed in the municipalities Balancán and Tenosique in the east of Tabasco (17°15′–18°10′N, 91°01′–91°46′W) (Fig. 1) comprising an area of 5474 km$^2$. The region is characterized by a warm (mean annual temperature 26 °C) and humid climate with precipitation throughout the whole year (1750 mm annual precipitation) (INEGI, 2001). The region is mainly a plain (67% of area, elevation <20 m a.s.l.) with hills (29%, 20–200 m a.s.l.) and mountains (4%, max. 640 m a.s.l.) located in the southern part. The dominant soils are Gleysols over alluvial sediments (plain), Vertisols, Cambisols, Luvisols and Acrisols over Miocene or Oligocene sediments (hills) and Leptosols and Regosols over limestone (mountains) (INEGI, 1985). The two municipalities have suffered dramatic changes of land cover during the last 60 years. While in 1950 the very sparsely populated region still was dominated by various types of tropical rainforest, by 2003 only about 10% of forests (including secondary forests) were left (Isaac-Márquez, 2008), mainly in the less accessible mountainous areas. Due to governmental programs of subsidies for cattle ranching, the percentage of grassland has increased to 78% (Casco, 1980; Tudela, 1989; Isaac-Márquez et al., 2008). Annual and perennial crops cover approximately 4% of the study region; the remainder consists mainly of wetlands and temporarily flooded areas.

Each site corresponded to a plot of homogenous land use. Four domain experts of forestry (SO-G, BdJ[1]), cattle-breeding (SH-D), agriculture (EH-L) and pedology (VG) had the task to evaluate land use on these sites. In particular, forested plots were evaluated with regard to structure, composition and perturbations, grazing cattle and agricultural plots with regard to the ecological sustainability of the actual site management, and soils on all plots with regard to soil fertility. For ease of communication, we refer to each of the four evaluations as an index of ecological condition in the remainder of this paper.

Based on personal knowledge and experience, each expert chose a number of primary indicators that in his or her opinion would satisfy the purpose of evaluating ecological condition. Their values were determined in the field between March and December 2004. Non-numeric primary indicators were scaled from 0 to 1,

[1] Author initials.

Author's personal copy



**Table 1**
Structure of rule-based and fuzzy-rule based aggregation of primary indicators *A* and *B* to intermediate variable *X*.

| Rule | Rule-based | | | Fuzzy rule-based | | |
|---|---|---|---|---|---|---|
| | if $A$ = | and if $B$ = | then $X$ = | if $A$ = | and if $B$ = | then $X$ = |
| 1 | $a_1$ | $b_1$ | $x_1$ | Low | Low | Low, medium or high |
| 2 | $a_1$ | $b_2$ | $x_2$ | Low | Medium | Low, medium or high |
| 3 | $a_1$ | $b_3$ | $x_3$ | Low | High | Low, medium or high |
| 4 | $a_2$ | $b_1$ | $x_4$ | Medium | Low | Low, medium or high |
| 5 | $a_2$ | $b_2$ | $x_5$ | Medium | Medium | Low, medium or high |
| 6 | $a_2$ | $b_3$ | $x_6$ | Medium | High | Low, medium or high |
| 7 | $a_3$ | $b_1$ | $x_7$ | High | Low | Low, medium or high |
| 8 | $a_3$ | $b_2$ | $x_8$ | High | Medium | Low, medium or high |
| 9 | $a_3$ | $b_3$ | $x_9$ | High | High | Low, medium or high |

where 0 represented "disastrous" and 1 represented "excellent". Based on the primary indicators and expertise, each domain expert evaluated ecological condition on each site on a scale from 0 to 1 (0: very bad; 1: very good).

Subsequently, each domain expert was asked to represent his reasoning in an explicit model, supported by a knowledge-engineer who himself is an ecologist (CK[1]). The experts should mould their ways of evaluating the sites into a hierarchical structure, aggregating two or more primary indicators at a time to higher level intermediate variables, and finally aggregating the intermediate variables into an index of ecological condition. The hierarchical structure was chosen (a) in order to support the domain experts in structuring their ecological reasoning, (b) in order to reduce rule-complexity at each aggregation level (see below). The methods applied in indicator aggregation were:

(a) simple mathematical algorithms (average, weighed average, minimum, maximum). For example, if primary indicator $A = a$ and primary indicator $B = b$, then the value $x$ of the intermediate variable $X$ is determined as $x = (a + b)/2$ or as $x = (w_1*a + w_2*b)/(w_1 + w_2)$, where $w_1$ and $w_2$ are weights, or as $x = \min(a, b)$ or $x = \max(a, b)$.
(b) a set of IF-THEN rules in the case of non-linear interactions between two or more indicators at an ordinal scale. For example, if primary indicator A can have the discrete values $a_1$, $a_2$ and $a_3$, and primary indicator $B$ can have the discrete values $b_1$, $b_2$ and $b_3$, then the value $x$ of the intermediate variable $X$ is determined by a set of nine rules (Table 1).
(c) a fuzzy rule-based model in the case of non-linear interactions between two or more indicators at a continuous numerical scale or at an ordinal scale with a large number of possible values. For example, if both primary indicators $A$ and $B$ can have numerical values from 0 to 1, then the value $x$ of the intermediate variable $X$ is determined by a set of rules and by fuzzy sets representing the linguistic variables "low A", "medium A", "high A", "low B", "medium B", "high B" as well as the output "low X", "medium X", "high X" (Fig. 2, Table 1).

While in classic set theory an object can only either be a member (membership = 1) or not be a member (membership = 0) of a given set, the central idea of fuzzy set theory is that a member of a set may have partial membership, which consequently may possess all possible values between 0 and 1. The closer the membership of an element is to 1, the more it belongs to the set; the closer the membership of an element is to 0, the less it belongs to the set. The value $a = 0.4$ of primary indicator $A$ in Fig. 2, for example, has higher membership in the set "$a$ is medium" than in the set "$a$ is low"; it does not belong at all to the set "$a$ is high". The determination of the model output consists of three steps: First, the observed values of the primary indicators are translated into membership values in the fuzzy sets (called *fuzzification*); second, the memberships of the consequence of the applying rules in the fuzzy sets of the intermediate variable $X$ are calculated (called *fuzzy inference*); third, the fuzzy result is converted into a discrete numerical output (called *defuzzification*) (see Bothe, 1995 or Zimmermann, 1996 for an introduction to fuzzy models). Fuzzy rule-based models have become comparably popular in ecological modelling (Li and Rykiel, 1996; Salski, 1996) and there exist various examples in the context of evaluation, bioindication and sustainable management (for example, Mendoza and Prabhu, 2003; Kampichler and Platen, 2004).

The hierarchical structure of the model helps to keep the rule-bases as simple as possible. Assume three primary indicators, $A$, $B$ and $C$, with three levels each, that are to be aggregated to an index $I$: direct aggregation needs $3^3 = 27$ rules with three antecedents each, whereas the aggregation of $A$ and $B$ to intermediate variable $X$ and the aggrgeation of $X$ and $C$ to index $I$ requires only $3^2 + 3^2 = 18$ rules with only two antecedents each.

The expert evaluations of soil quality and ecological condition of forest, agricultural and cattle-ranching systems were used as training cases in order to induce regression trees (Breiman et al., 1998) using the primary indicators as independent variables and expert evaluation as the dependent variable. Regression trees are based on the assumption that the relationship between independent and dependent variables is not constant over the entire range

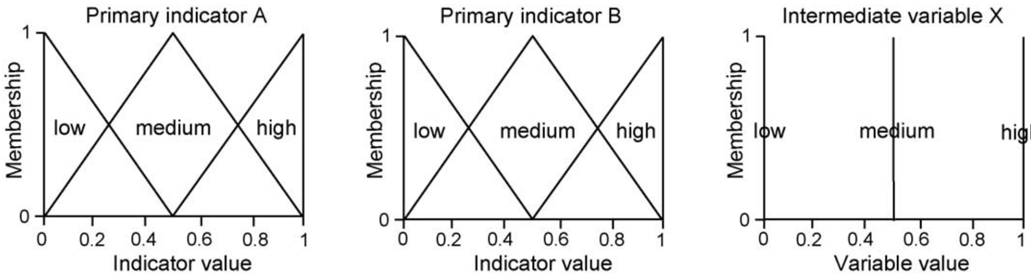


**Fig. 2.** Example of fuzzy sets. Primary indicators *A* and *B* are structured into three triangular sets each, representing "low", "medium" and "high" values; the values "low", "medium" and "high" of output variable *X* are represented by singletons.

Author's personal copy



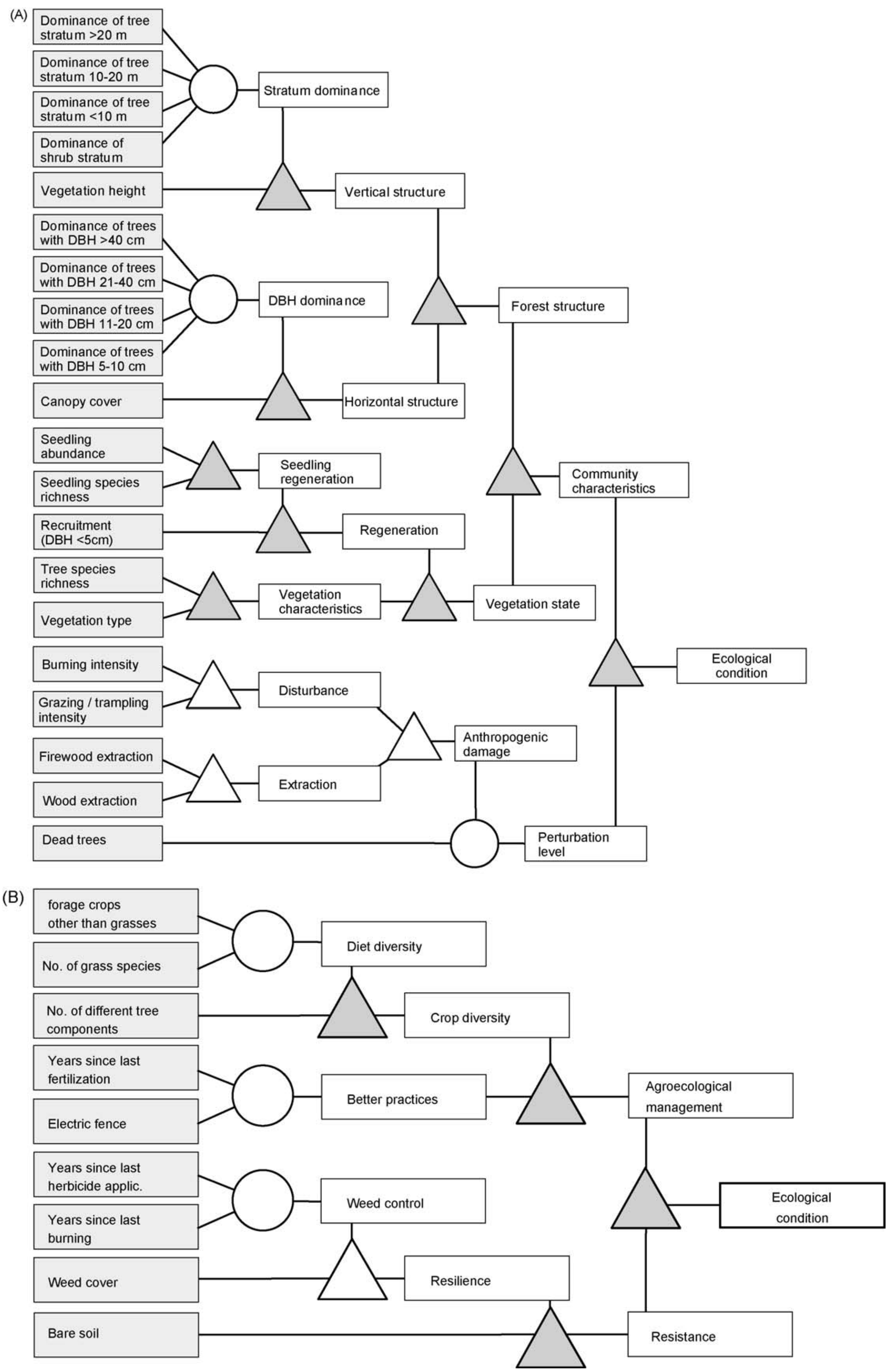


**Fig. 3.** Model structure for the evaluation of ecological condition of three land-use systems and the soil developed by domain experts with the help of an knowledge-engineer: forest systems (A), cattle breeding systems (B), agricultural systems (C), soil (D). Primary indicators are shaded in grey. Circles represent simple mathematic algorithms, white triangles represent rule sets, and grey triangles represent rule sets based on fuzzy logic.

Author's personal copy



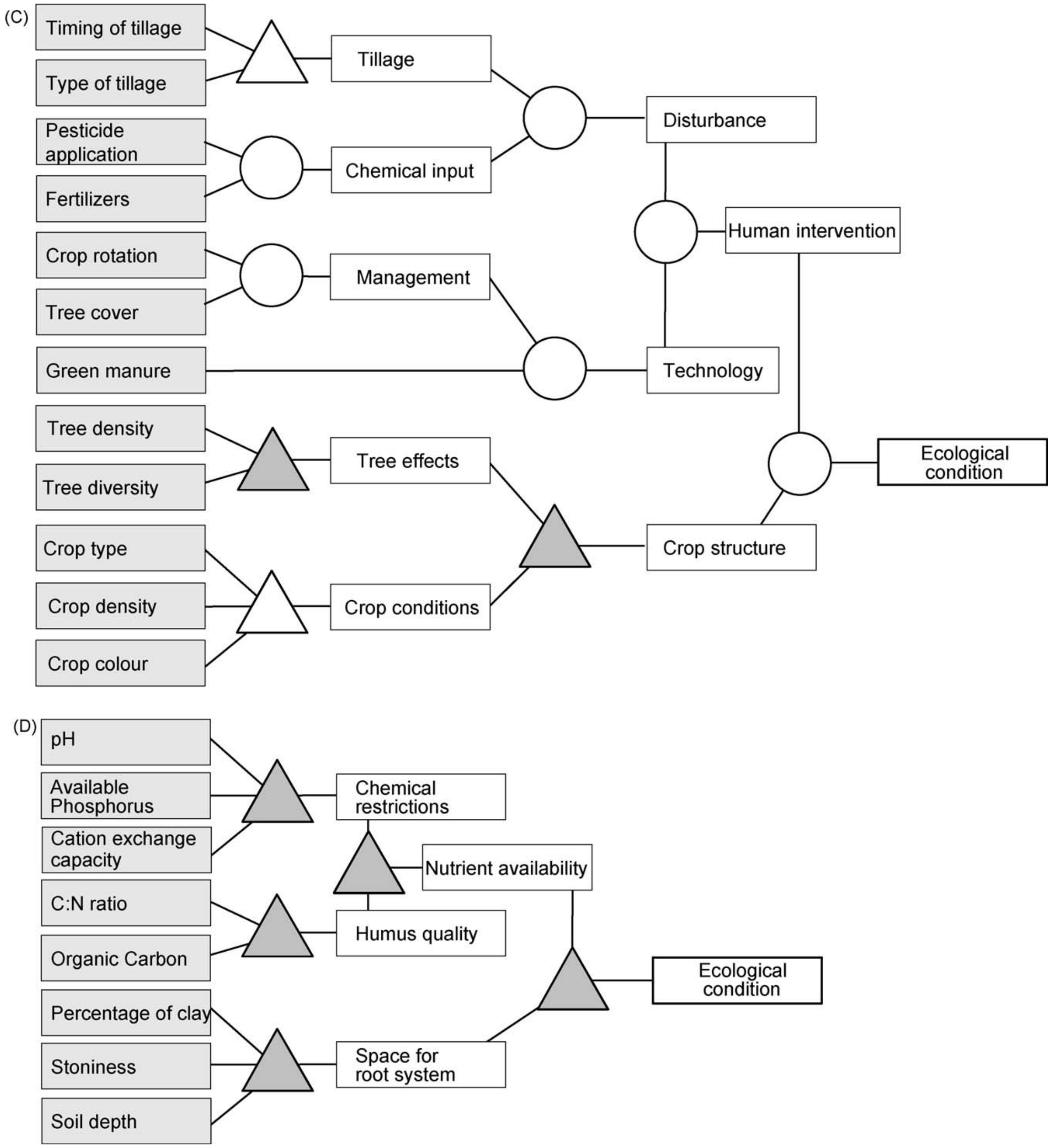


Fig. 3. (*Continued*).

of possible variable values, but can be approximated in smaller subdomains. Regression trees are thus constructed iteratively splitting the data into homogeneous subsets, which results in a tree-like structure where each branch is defined by a certain range of values of the independent variables and the terminal nodes of the tree consist of a predicted constant. Regression trees are ideally suited for exploring data that exhibit non-linearity and high-order interactions; in contrast to classification trees they are, however, only occasionally used in ecological data analysis (for example, Andersen et al., 2000; De'ath and Fabricius, 2000; Kampichler et al., 2000; Džeroski and Drumm, 2003).

Personal expert evaluations and model outputs were compared by calculating Spearman rank correlations as well as their mean absolute deviations (MAD) calculated as

$$MAD = (\Sigma \quad |x_{i,expert} - x_{i,model}|)/n$$

where $x_{i,expert}$ is the ecological condition of the $i$th site as evaluated by the expert domain, $x_{i,model}$ is the ecological condition of the $i$th site as predicted by the model, and $n$ is the number of sites. Personal expert evaluations and the regression trees were compared in the same way. All statistical analyses were performed with R (R Development Core Team, 2007); for

Author's personal copy



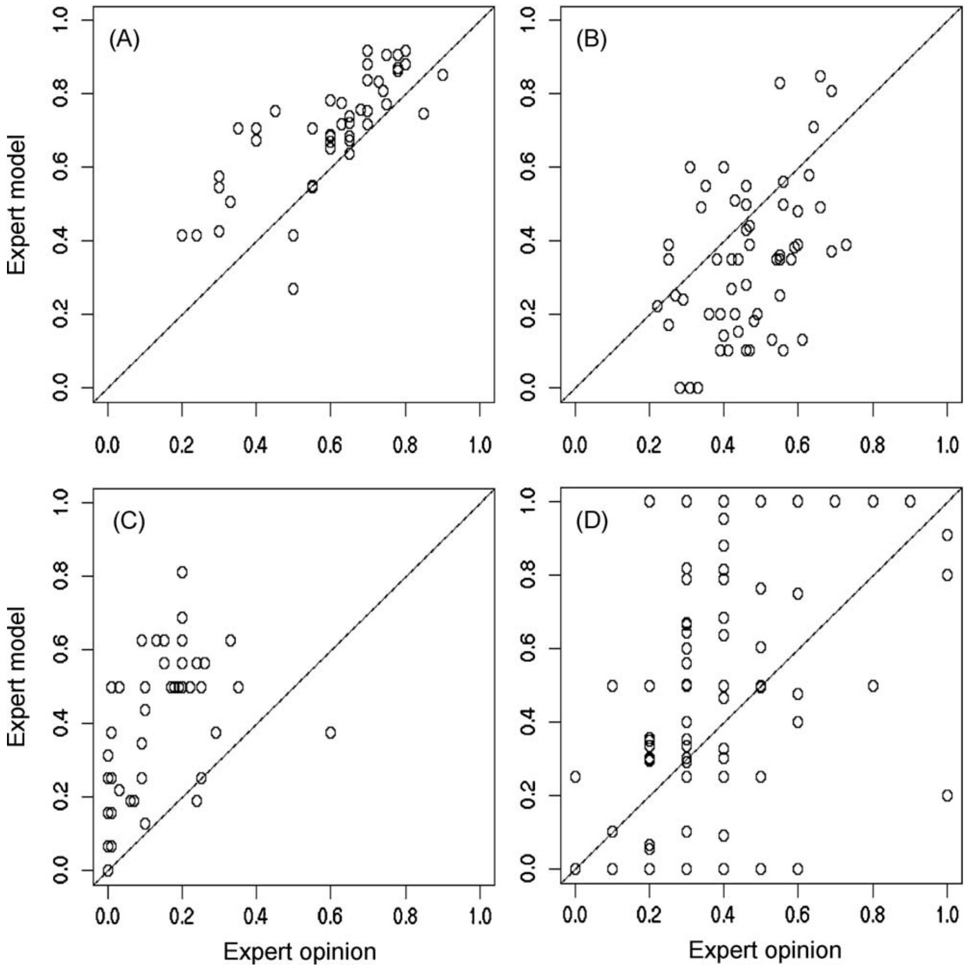


**Fig. 4.** Comparison between the expert evaluations of the ecological condition of three land-use systems and the soil and the output of the correspondent models developed by domain experts: Forest systems (A), cattle ranching systems (B), agricultural systems (C), soil (D). Ecological condition is scaled between 0 (very bad) and 1 (very good).

regression tree induction we used the R package tree (Ripley, 2007).

## 3. Results

The domain experts chose between 8 (soils) and 20 (primary and secondary forests) primary variables for their personal evaluations of the sites. They aggregated the primary variables to an index of ecological condition by means of between 5 (soils) and 15 (primary and secondary forests) nodes generating between 4 and 14 intermediate variables (Fig. 3). Detailed descriptions of each model (justification for variable choice and variable aggregation, specific algorithms used in each model node, rule sets, fuzzy sets) will be published elsewhere (Hernández-Daumás et al., submitted for publication; Ochoa-Gaona et al., submitted for publication).

There were noteworthy differences between the experts' opinions and the outputs of their models (Fig. 4). The Spearman correlation coefficient could be as low as 0.417 and the mean absolute deviation between expert opinion and the ecological condition index as calculated by the model attained values as high as 0.279 (Table 2).

All regression trees (Fig. 5) included considerably less primary variables than chosen by the domain experts. Numbers of variables used dropped from 20 to 5 (primary and secondary forests), from 9 to 5 (cattle ranching), from 12 to 4 (agriculture) and from 8 to 5 (soils) (Table 2). Nonetheless, there was higher correspondence between expert opinion and regression trees (Fig. 6) than between expert opinion and their own models with considerable higher correlation coefficients and considerable lower MADs (Table 2).

**Table 2**
Comparison between expert opinion and expert model. All Spearman rank correlation coefficients are statistically significant at $p < 0.001$.

| Knowledge domain | Comparison between expert opinion and expert model | | Comparison between expert opinion and regression tree | | Variable reduction by regression tree |
|---|---|---|---|---|---|
| | $r_{Spearman}$ | MAD[a] | $r_{Spearman}$ | MAD[a] | |
| Primary and secondary forests | 0.825 | 0.126 | 0.942 | 0.044 | 75% |
| Cattle-breeding | 0.417 | 0.188 | 0.842 | 0.052 | 44% |
| Agriculture | 0.591 | 0.272 | 0.746 | 0.057 | 67% |
| Soil | 0.495 | 0.279 | 0.901 | 0.069 | 38% |

[a] MAD, mean absolute deviation.

Author's personal copy



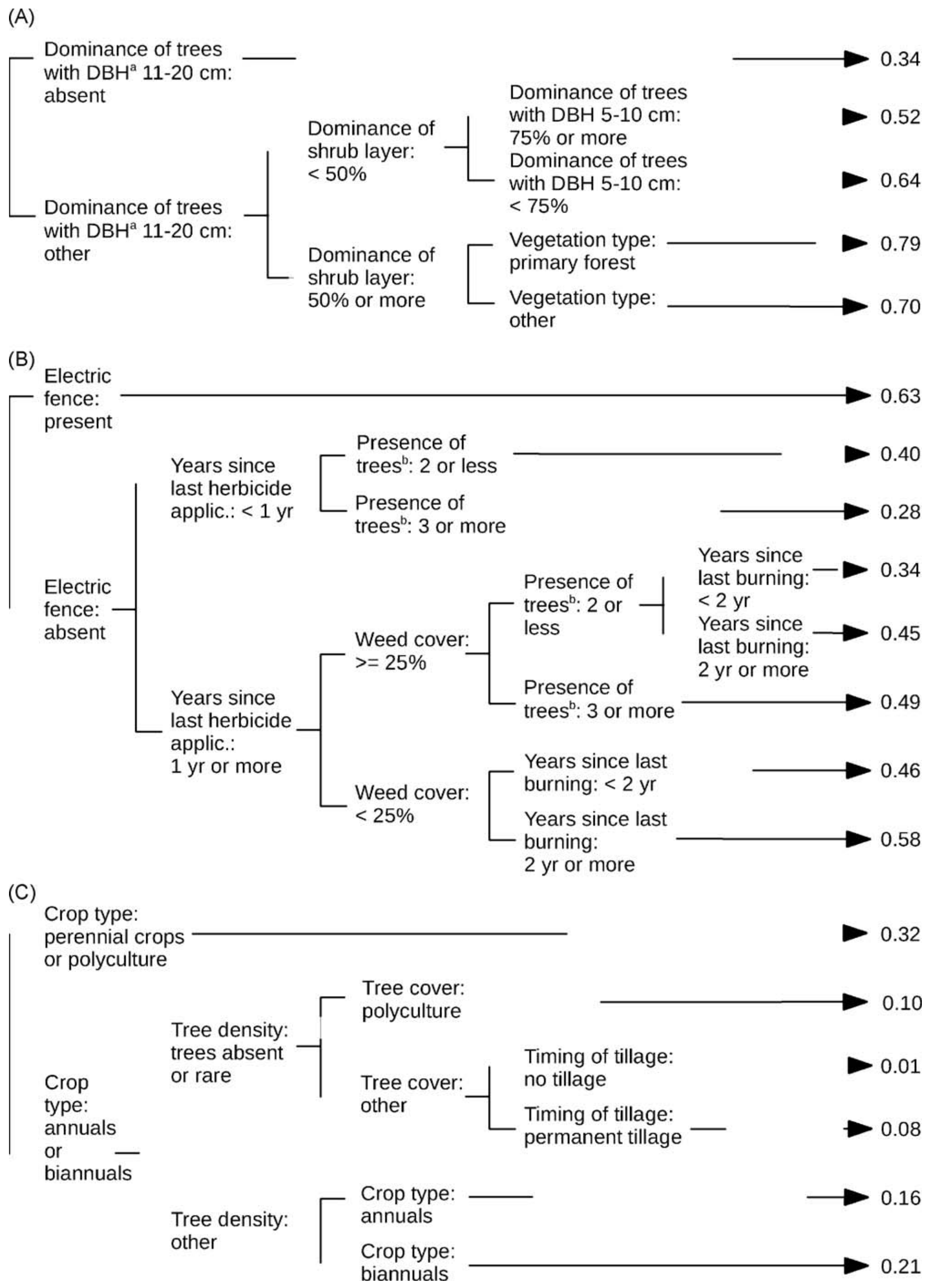


**Fig. 5.** Regression trees for the evaluation of ecological condition of three land-use systems and the soil trained by the expert evaluation results: Forest systems (A), cattle ranching systems (B), agricultural systems (C), soil (D). Ecological condition is scaled between 0 (very bad) and 1 (very good). [a]DBH, diameter in breast height; [b]relative value ranging from 0 to 5 considering presence of at least two types from (i) scattered, (ii) riparian, (iii) live fences and (iv) other important trees, one of them has to be (i) or (ii) in a well developed canopy; [c]CEC, cation exchange capacity.

## 4. Discussion

Human experts are human beings, and as with all humans, their decisions are influenced by personal experience, intuition, heuristics and bias. Thus, experts of the same knowledge domain often disagree on the relative importance of different evaluation criteria (Weisberg et al., 2008). Uncertainty or vagueness of how to rate the importance of every possible explanatory variable might motivate domain experts to choose a high number of primary indicators, obviously obeying a rule of thumb such as “better measure a few variables too many than to miss an important factor”. Regression trees reduced the number of primary indicators considerably, at least by 38%. Since expert opinion served as database for their training, regression trees yielded results corresponding closely to the expert’s evaluation but were more parsimonious in terms of necessary variables. Thus, the automated induction of regression trees seems to be a promising way to develop indicators for narrowly defined ecological assessments as needed for decision-making at the local or regional scale. A two-stage procedure – first stage: primary indicator selection by a local domain expert, preliminary sampling, expert evaluation, induction of a regression-tree trained by the expert evaluation results; second stage: comprehensive sampling taking into account only the narrowed indicator set – could reduce considerably the time

Author's personal copy



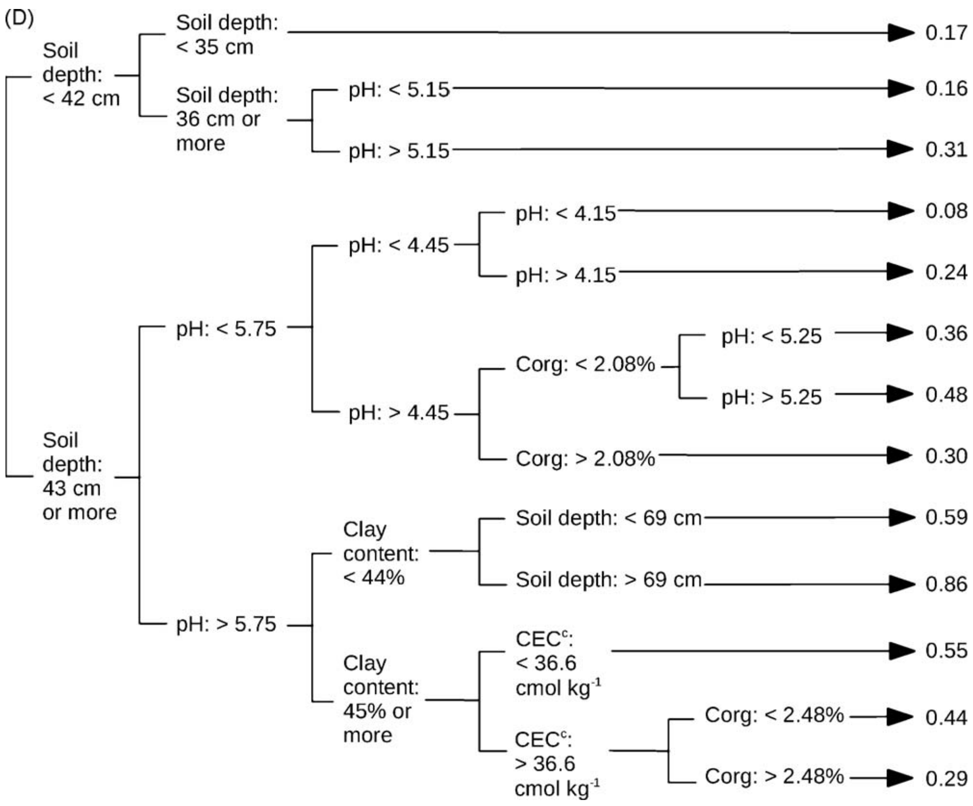


Fig. 5. (*Continued*).

spent in the field for sampling and data acquisition. A reduction of variables by 75% such as in the case of primary and secondary forests could allow for the multiplication of sample size without increasing project expenditure, thus producing more data and higher statistical power.

Model development by the domain experts themselves obviously had a serious drawback: in all cases, the model outputs were not as close to their personal evaluations as were the results of regression tree induction. Initial expert doubts on the validity of the trees could easily be resolved. For example, the expert on cattle-ranching systems observed the condensation of the values of the ecological condition index to a narrower range from [0.22 0.73] in the expert's evaluation to [0.28 0.63] in the regression tree (Fig. 6B). This reflects the problem of assigning values to a few extreme plots (very good and very bad ones); while the tree yields values that corresponded well with the experts evaluation, the experts evaluation and the model did not as shown by their quartiles (expert evaluation: 0.39, 0.46, 0.56; expert model: 0.20, 0.35, 0.49; regression tree: 0.40, 0.46, 0.58).

Moreover, machine learning methods have the potential to improve ecological knowledge. Džeroski et al. (1997), for example, reported how classification tree induction enhanced knowledge on the role of several bioindicator taxons in Slovenian rivers. The experts' opinions on the regression trees generated in this study were conflicting. Some aspects of the trees were positively recognised, others caused contradiction. For example, the forest experts acknowledged the primary bifurcation in the corresponding regression tree (Dominance of trees with diameter in breast height >11–20 cm; Fig. 5A), but doubted the validity of the second bifurcation (shrub layer <50% vs. >50%). Also, the expert on cattle-ranching systems was surprised by the fact that the sole presence of electric fences should be sufficient to qualify a plot as comparably good (index of ecological condition = 0.63, see Fig. 5B). This conflict most probably arises due to the fact that experts intent to find relationships between primary variables and ecological condition which are generally valid and not restricted to the study area, whereas tree induction yields relationships valid specifically for the training set of cases. So while the dominance of the shrub layer is generally not a valid approach to distinguish between better and worse ecological condition (for example, mature rainforests with a high and closed canopy normally have a little developed shrub layer, so a lack of the shrub layer does not always mean low quality), it may well be so for the restricted number of observations made in the study area. Likewise, the presence of electric fences is not a necessary trait of ecologically sound cattle-ranching systems; in the study area they might be a valuable indicator of the application of best practices in rangeland management. This underpins the potential of rapidly developed regression trees for variable selection and minimisation of time and money expenditure for sampling at the local or regional scale; however, for the development of generalisable models, expert knowledge and experience is indispensable (except when a huge database collected at a larger scale should be available).

The standard for the evaluation of the experts' models and the regression trees in this study were the personal evaluations made by the experts themselves. Experts may be wrong, however, and there exists the possibility that their models, based on explicit reasoning, reflect the situation in the real world better than their synthetic expert opinion does which might be susceptible to subjectivity and bias. The suggested procedure "expert evaluation – regression-tree induction – sampling based on narrowed indicator-set" could be made more robust by consulting a group of local experts and applying Delphi methodology (Bowles, 1999; Rowe and Wright, 1999; Linstone and Turoff, 2002) in the stages of primary variable selection and personal plot evaluation; regression-trees based on personal evaluation averaged across a number of experts will suffer less danger of being biased due to expert

Author's personal copy



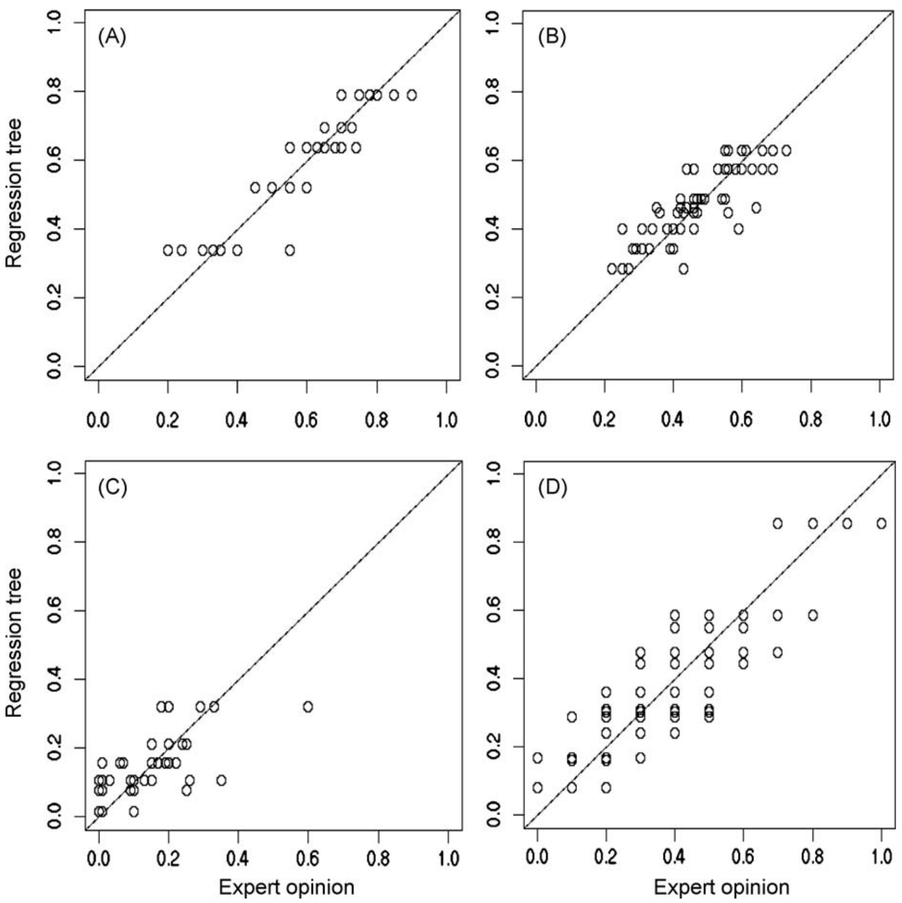


**Fig. 6.** Comparison between the expert evaluations of the ecological condition of three land-use systems and the soil and the output of the correspondent regression trees trained by the expert evaluation results: Forest systems (A), cattle breeding systems (B), agricultural systems (C), soil (D). Ecological condition is scaled between 0 (very bad) and 1 (very good).

subjectivity which as we know can be considerable (Weisberg et al., 2008).

## 5. Conclusions

Knowledge engineering, i.e., the conversion of expert opinion into a formal model of decision making, was tedious and time consuming in comparison with the knowledge discovery approach, i.e., the automated induction of regression-trees. Moreover, the correspondence between regression tree output and expert opinion was considerably higher than the correspondence between expert opinion and the experts' own models, despite the trees used being only a subset of the primary variables. The application of regression tree induction, thus, has a high potential for the rapid development of indicators for narrowly defined ecological assessments as needed for decision-making at the local or regional scale and for the minimisation of sampling effort due to variable space reduction. Expert knowledge will still be indispensable for the development of generalisable models valid at larger spatial scales.

## Acknowledgements

This study was funded jointly by SEMARNAT (Mexican Ministry for Natural Resources Management) and CONACyT (Mexican National Council for Science and Technology) (Project No. SEMARNAT-2002-C01-1109). We thank Corinna Hadaway (Edinburgh) for linguistic revision of the manuscript.

## References

Andersen, M.C., Watts, J.M., Freilich, J.E., Yool, S.R., Wakefield, G.I., McCauley, J.F., Fahnestock, P.B., 2000. Regression-tree modeling of desert tortoise habitat in the Central Mojave desert. Ecological Applications 10, 890–900.

Bothe, H.-H., 1995. Fuzzy Logic, 2nd ed. Springer, Berlin, 255 pp.

Bowles, N., 1999. The Delphi technique. Nursing Standard 13, 32–36.

Breiman, L., Friedman, J.H., Olshen, R.A., Stone, C.J., 1998. Classification and Regression Trees, 1st reprint. Chapman & Hall/CRC, Boca Raton, 358 pp.

Büchs, W., 2003. Biodiversity and agri-environmental indicators—general scopes and skills with special reference to the habitat level. Agriculture, Ecosystems & Environment 98, 35–78.

Casco, R., 1980. Los planes de desarrollo del trópico: el caso de Balancán-Tenosique, Tabasco. Centro de Ecodesarrollo, Mexico, 72 pp.

Challenger, A., 1998. Utilización y Conservación de los Ecosistemas Terrestres de México. CONABIO-UNAM-Sierra Madre, Mexico, 847 pp.

De'ath, G., Fabricius, K.E., 2000. Classification and regression trees: a powerful yet simple technique for ecological data analysis. Ecology 81, 3178–3192.

Džeroski, S., Drumm, D., 2003. Using regression trees to identify the habitat preference of the Sea Cucumber (*Holothuria leucospilota*) on Rarotonga, Cook Islands. Ecological Modelling 170, 219–226.

Džeroski, S., Grbović, J., Walley, W.J., Kompare, B., 1997. Using machine learning techniques in the construction of models. II. Data analysis with rule induction. Ecological Modelling 95, 95–111.

Finlay, J., Dix, A., 1996. An Introduction to Artificial Intelligence. UCL Press, London, 276 pp.

Gamborg, C., Sandøe, P., 2005. Sustainability in farm animal breeding: a review. Livestock Production Science 92, 221–231.

Hernández-Daumás, S., Kampichler, C., Ochoa-Gaona, S., Geissen, V., Huerta-Lwanga, E., de Jong, B., submitted. An index for the evaluation of the ecological condition of cattle ranching systems in the neotropics. Journal of Agricultural Science.

Hurni, H., 2000. Assessing sustainable land management (SLM). Agriculture, Ecosystems & Environment 81, 83–92.

INEGI, 1985. Carta Edafológica, Villahermosa E15-8, 1: 250,000. Instituto Nacional de Estadística, Geografía e Informática, Aguascalientes, Mexico.

Author's personal copy



INEGI, 2001. Carta Estatal de Climas Tabasco 1: 1,000,000, 2nd ed. Instituto Nacional de Estadística, Geografía e Informática, Aguascalientes, Mexico.

Isaac-Márquez, R., 2008. Análisis del Cambio de Uso y Cobertura del Suelo en los Municipios de Balancán y Tenosique, Tabasco, México. Doctoral Thesis, El Colegio de la Frontera Sur, Villahermosa, ix+144 pp.

Isaac-Márquez, R., de Jong, B., Eastmond, A., Ochoa-Gaona, S., Hernández, S., Sandoval, J.L., 2008. Programas gubernamentales y respuestas campesinas en el uso del suelo: el caso de la zona oriente de Tabasco, México. Región y Sociedad 20 (43), 97–129.

Kampichler, C., Platen, R., 2004. Ground beetle occurrence and moor degradation: modelling a bioindication system by automated decision-tree induction and fuzzy logic. Ecological Indicators 4, 99–109.

Kampichler, C., Džeroski, S., Wieland, R., 2000. Application of machine learning techniques to the analysis of soil ecological data bases: relationships between habitat features and Collembola community characteristics. Soil Biology & Biochemistry 32, 197–209.

Karlen, D.L., Tomer, M.D., Neppel, J., Cambardella, C.A., 2004. A preliminary watershed scale soil quality assessment in north central Iowa, USA. Soil and Tillage Research 99, 291–299.

Li, B.-L., Rykiel, Jr., E.J., 1996. Introduction. In: Li, B.-L., Rykiel, Jr., E.J. (Eds.). Fuzzy Modelling in Ecology. Ecological Modelling, vol. 90. pp. 109–110.

Linstone, H. A. and Turoff, M., 2002. The Delphi Method: Techniques and Applications, Online Reproduction. Addison-Wesley, Reading, 618 pp. http://www.is.njit.edu/pubs/delphibook/.

Marchini, A., Facchinetti, T., Mistri, M., 2009. F-IND: a framework to design fuzzy indices of environmental conditions. Ecological Indicators 9, 485–496.

Medsker, L., Liebowitz, J., 1994. Design and Development of Expert Systems and Neural Networks. Macmillan College Publishing Company, New York, 273 pp.

Mendoza, G.A., Prabhu, R., 2003. Fuzzy methods for assessing criteria and indicators of sustainable forest management. Ecological Indicators 3, 227–236.

Ochoa-Gaona, S., Kampichler, C., de Jong, B.H.J., Hernández, S., Geissen, V., Huerta, E., submitted. A multi-criterion index for the evaluation of local tropical forest conditions in Mexico. Forest Ecology and Management.

OECD, 2008. Key Environmental Indicators. OECD Environment Directorate, Paris, 38 pp.

R Development Core Team, 2007. R: A Language and Environment for Statistical Computing. R Foundation for Statistical Computing, Vienna, Austria. ISBN 3-900051-07-0, URL: http://www.r-project.org.

Ripley, B., 2007. tree: Classification and Regression Trees. R Package Version 1.0-26. http://cran.r-project.org/.

Rowe, G., Wright, G., 1999. The Delphi technique as a forecasting tool: issues and analysis. International Journal of Forecasting 15, 353–375.

Salski, A., 1996. Introduction. In: Salski, A. (Ed.). Fuzzy Logic in Ecological Modelling. Ecological Modelling, vol. 85, pp. 1–2.

Sutherland, W.J., Pullin, A.S., Dolman, P.M., Knight, T.M., 2004. The need for evidence-based conservation. Trends in Ecology and Evolution 19, 305–308.

Tudela, F., 1989. La modernización forzada del trópico: el caso de Tabasco. El Colegio de México, Mexico, 391 pp.

Weisberg, S.B., Thompson, B., Ranasinghe, J.A., Montagne, D.E., Cadien, D.B., Dauer, D.M., Diener, D., Oliver, J., Reish, D.J., Velarde, R.G., Word, J.Q., 2008. The level of agreement among experts applying best professional judgement to assess the condition of benthic infaunal communities. Ecological Indicators 8, 389–394.

World Commission of Environment and Development, 1987. Our Common Future. Oxford University Press, Oxford, 416 pp.

Zimmermann, H.-J., 1996. Fuzzy Set Theory and its Applications, 3rd ed. Kluwer Academic Publishers, Boston, 435 pp.
